# Nanostructured Surfaces Enhance Nucleation Rate of Calcium Carbonate

**DOI:** 10.1002/smll.202402690

**Published:** 2024-08-20

**Authors:** Tobias Armstrong, Julian Schmid, Janne‐Petteri Niemelä, Ivo Utke, Thomas M. Schutzius

**Affiliations:** ^1^ Laboratory for Multiphase Thermofluidics and Surface Nanoengineering Department of Mechanical and Process Engineering ETH Zurich Sonneggstrasse 3 Zurich CH‐8092 Switzerland; ^2^ Laboratory for Mechanics of Materials and Nanostructures Empa – Swiss Federal Laboratories for Materials Science and Technology Feuerwerkerstrasse 39 Thun CH‐3602 Switzerland; ^3^ Department of Mechanical Engineering University of California Berkeley CA 94720 USA

**Keywords:** biomineralization, calcium carbonate, crystallization fouling, nanoscale engineering, nucleation, scalephobicity, surface nanostructure

## Abstract

Nucleation and growth of calcium carbonate on surfaces is of broad importance in nature and technology, being essential to the calcification of organisms, while negatively impacting energy conversion through crystallization fouling, also called scale formation. Previous work studied how confinements, surface energies, and functionalizations affect nucleation and polymorph formation, with surface‐water interactions and ion mobility playing important roles. However, the influence of surface nanostructures with nanocurvature—through pit and bump morphologies—on scale formation is unknown, limiting the development of scalephobic surfaces. Here, it is shown that nanoengineered surfaces enhance the nucleation rate by orders of magnitude, despite expected inhibition through effects like induced lattice strain through surface nanocurvature. Interfacial and holographic microscopy is used to quantify crystallite growth and find that nanoengineered interfaces experience slower individual growth rates while collectively the surface has 18% more deposited mass. Reconstructions through nanoscale cross‐section imaging of surfaces coupled with classical nucleation theory—utilizing local nanocurvature effects—show the collective enhancement of nano‐pits.

## Graphical abstract

In situ optical characterization demonstrates the substantial enhancing effects of nanoengineered surfaces at various supersaturations on calcium carbonate nucleation and growth. Nano‐pits with radii of mean curvature within one magnitude of the critical nucleus radius are identified as causing this effect. The findings provide insights for designing advanced functional surfaces, e.g. scalephobic surfaces, to control calcium carbonate crystallization.

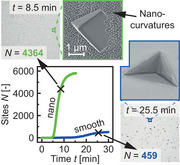

## 1 Introduction

Understanding and controlling calcium carbonate nucleation and growth is important in both nature and technology, exemplified by crystallization fouling in energy applications^[^ ^1^, ^2^, ^3^ ^]^ and the fabrication of advanced biomimicry materials, like nacre.^[^ ^4^, ^5^ ^]^ Previous work debates whether calcium carbonate nucleation mechanisms follow the classical nucleation theory (CNT) or non‐classical mechanisms.^[^ ^6^, ^7^, ^8^, ^9^ ^]^ Nucleating crystallites in CNT overcome their thermodynamic nucleation barrier at a critical radius through ion‐by‐ion attachment.^[^ ^5^, ^10^, ^11^, ^12^, ^13^, ^14^ ^]^ Non‐classical mechanisms have complex free energy landscapes, including multiple coexisting nucleation pathways,^[^ ^15^ ^]^ such as stable pre‐nucleation clusters,^[^ ^16^ ^]^ multiple polymorphs (e.g., amorphous calcium carbonate (ACC), calcite, etc.), and crystallization by particle attachment.^[^ ^4^, ^17^, ^18^ ^]^ Nucleation behavior is dependent on supersaturation,^[^ ^15^ ^]^ confinement,^[^ ^19^, ^20^, ^21^, ^22^ ^]^ and surface wetting by varying surface energy, functional group termination,^[^ ^5^, ^10^, ^11^, ^12^, ^13^, ^14^, ^23^, ^24^, ^25^, ^26^ ^]^ topography,^[^ ^27^, ^28^ ^]^ temperature,^[^ ^29^, ^30^ ^]^ and salinity.^[^ ^31^ ^]^ Further, complex systems with several interfaces^[^ ^32^, ^33^, ^34^ ^]^ and additives in the crystallization solution^[^ ^34^, ^35^ ^]^ impact the nucleation onset.

Surface energy and functional group termination have been shown to affect the nucleation behavior of calcium carbonate.^[^ ^23^ ^]^ Fluorinated and rare earth metal surfaces^[^ ^30^, ^36^, ^37^, ^38^, ^39^ ^]^ as well as functional groups with low affinity to calcium ions^[^ ^26^ ^]^ tend to inhibit nucleation while those with high affinity promote nucleation.^[^ ^5^, ^12^, ^13^, ^14^, ^23^, ^24^ ^]^ The concept is not unanimously accepted with reports of termination‐independent nucleation mechanisms.^[^ ^11^, ^40^ ^]^ The effect of surface roughness on calcium carbonate nucleation, as well as related minerals like calcium sulfate, is not clear, with previous work showing that it can promote,^[^ ^41^, ^42^, ^43^, ^44^, ^45^ ^]^ inhibit,^[^ ^27^, ^44^, ^46^, ^47^ ^]^ or has no effect.^[^ ^48^ ^]^ Previous work has established that surface nanocurvature^[^ ^49^, ^50^, ^51^ ^]^ can affect the nucleation behavior of ice,^[^ ^52^, ^53^ ^]^ aspirin,^[^ ^54^, ^55^ ^]^ indomethacin,^[^ ^55^ ^]^ and hydrogen^[^ ^56^ ^]^ as well as regulate nucleation pathways through matching or lattice mismatching between the surface nanotopography and crystallites.^[^ ^28^, ^52^, ^53^, ^55^, ^57^, ^58^ ^]^ However, the effect of surface nanocurvature and lattice mismatching^[^ ^59^ ^]^ on calcium carbonate nucleation and growth is not known but is necessary to understand individual crystallite nucleation and the formation of more complex deposits and structures as found in nature and technology.

Here we investigate experimentally and theoretically the combined effects of surface nanocurvature and supersaturation on calcium carbonate nucleation and growth from aqueous supersaturated solution, identify a nucleation‐enhancing mechanism, and interpret the effect of this enhancement on the collective evolution toward more complex deposits. We report that both nanoengineered and smooth surfaces with the same surface functionalization preparation show nucleation of expected calcite formation on carboxylate surfaces. We observe no other polymorphs, even though the nanocurvature on the nanoengineered surface mismatches the calcite crystal lattice. The nanoengineered surfaces show an enhancement of nucleation rates by more than one order of magnitude. Simultaneous brightfield, total internal reflection fluorescence (TIRF), and digital holography microscopy (DHM) were used to investigate individual crystallite nucleation and growth, and we reveal that surface nanocurvature leads to enhanced nucleation rates, with a critical crystallite separation distance resulting in a cross‐over to growth, affecting the evolution of more collective surface deposits. This characterization was made possible through a trained instance segmentation algorithm and the crystallite volume through phase shift reconstruction of the acquired hologram. Through this, we were able to show that nanoengineered surfaces can regulate both kinetic and thermodynamic nucleation barriers. Spatial reconstruction of the surface topographies and subsequent mapping of local thermodynamic nucleation barriers at the nanoscale with classical nucleation theory supports our experimental findings. We expect that the importance of nanocurvature for calcium carbonate crystallization will guide the design of advanced functional surfaces including scalephobic ones.

## 2 Results and Discussion

**Figure 1:**
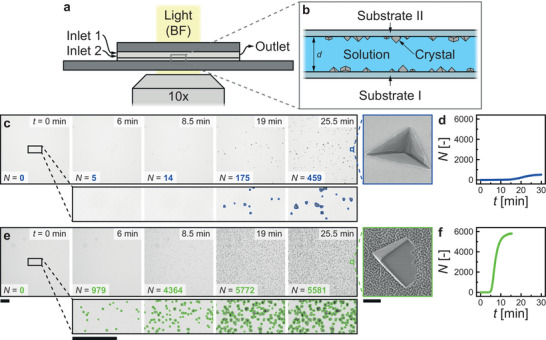
Effect of a nanoengineered surface on calcium carbonate nucleation and growth. a) The side‐view schematic shows a microfluidic cell on an optical light microscope, having two inlets—one for calcium chloride solution and one for sodium carbonate solution—and one outlet for the mixed solution. When the solutions mix, they become supersaturated (*σ* > 0; in this study, *σ* = 4.44–4.86). b) Schematic showing the observation zone after mixing with two substrate windows (I: bottom substrate; II: top substrate), which are in contact with the laminar (height *d* = 0.8 mm; width of *w* = 1.7 mm; volume flow $\dot{V}$ = 500 µL min^−1^; $Re=\frac{2\rho \dot{V}}{\eta \left(d+w\right)}$ < 10) supersaturated aqueous solutions. Image sequences showing calcium carbonate nucleation and growth on c) smooth (blue) and e) nanoengineered (green) surfaces as bottom substrate I (*σ* = 4.62). The number of detected crystallites in each image is shown, *N*. The crystallite outline is shown for the magnified image sequence. Inset images show micrographs of the crystallites on the smooth and nanoengineered surfaces. d), f) Plot of the number of crystallites *N* versus time *t* for the c) smooth and e) nanoengineered surface. Scale bars: c), e) 100 µm; Inset: 500 nm.

To understand the influence of surface nanotopography on calcium carbonate nucleation and growth in aqueous supersaturated solutions, we generated smooth and nanoengineered surfaces on precision cover glass substrates, enabling the necessary optical access. The nanotopography consisted of a hexagonal nanopattern array of glass nano‐bumps (height ≈80 nm), which were generated by block copolymer lithography, sequential infiltration, and dry etching (Section S1, Supporting Information). Both smooth and nanoengineered surfaces were then passivated with silicon dioxide (SiO_2_), silanized, coated with evaporated gold, and functionalized by a thiol‐based carboxy‐terminated self‐assembling monolayer (SAM). By using the same thiol‐based SAM surface preparation for smooth and nanoengineered surfaces, we ensure chemical defect comparability^[^ ^60^ ^]^ and isolate the nano‐bump effect on nucleation. The carboxyl‐termination is crucial for calcite formation through high affinity to calcium ions. To determine the effect of supersaturation, *σ*, and flow rate, $\dot{V}$, on the experimentally measured nucleation rate, *J*, separate aqueous solutions of calcium chloride and sodium carbonate were mixed in a microfluidic flow chamber at temperature, *T* = 22.5 °C, producing uniform supersaturated solutions over our test surfaces, $\sigma \;=\;\ln[\left(a_{Ca^{2+}}\right)\left(a_{{\mathrm{CO}}_{3}^{2-}}\right)/K_{\mathrm{sp}}]$ where *a_i_* are activities of ions *i* and *K*_sp_ is the solubility product of the forming phase (Section S2, Supporting Information). The width and height of the flow channel are *w* and *d*, respectively, which for a given total flow rate, $\dot{V}$, yields the mean flow velocity, $\bar{v}=\frac{\dot{V}}{dw}$ and Reynolds number $Re=\frac{2\rho \dot{V}}{\eta \left(d+w\right)}$, **Figure** **1**a,b. The microfluidic cell design ensures full mixing of the solution in a mixing zones, a large surface area to volume ratio, and a consistent advection‐driven supply of the chosen *σ*, which is crucial for controlling and determining the nucleation behavior. The observation zone geometry in Figure 1b is large enough to prevent confinement,^[^ ^19^, ^20^, ^21^, ^22^ ^]^ and due to the advection‐driven supply of the bulk supersaturation, the two substrate positions do not influence each other (Section S3, Supporting Information). Figure 1c,e show image sequences of calcium carbonate crystallites heterogeneously nucleating on smooth and nanoengineered surfaces, respectively, in an environment of *σ* = 4.62 with respect to calcite and *Re* ≈7 (Movies S1 and S2, Supporting Information). All results related to smooth surfaces are color‐coded in blue, while everything related to nanoengineered surfaces is color‐coded in green. This color code applies to all figures. The period before the number of detected crystallites on the surface, *N*, grows linearly with time, *t*, is defined as the induction time. The linear increase of independent nucleation events determines the experimental nucleation rate, *dN*/*dt*, Figure 1d,f. After *N* begins to diverge from the linear slope, subsequent nucleation events are no longer independent.^[^ ^12^ ^]^ This is due to local crystallite effects in the boundary layer and is independent of the counter‐surface through the advection‐dominated bulk flow in the observation zone channel (Péclet number, *Pe* ≫ 1) (Section S4, Supporting Information). For the smooth surface, we observe *dN*/*dt* ≈52 min^−1^, while for the nanoengineered surface, we observe *dN*/*dt* ≈1534 min^−1^. This increase in *dN*/*dt* by more than one order of magnitude occurs although the surface area of the nanoengineered surface due to its Wenzel roughness,^[^ ^61^ ^]^ *r*_W_, is only *r*_W_ = 2.45 times larger than the smooth surface, for the length scale of the nanoengineered features, indicating that the enhancement cannot be due to an increase in surface area alone. Nucleation enhancement due to surface nanocurvature has been shown for other materials, such as ice,^[^ ^52^, ^53^ ^]^ and is rationalized by classical nucleation theory.

**Figure 2:**
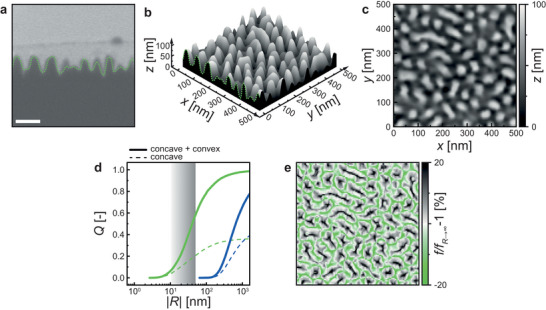
Quantification of nanotopography and its influence on the geometric factor f, influencing the thermodynamic nucleation barrier. a) Tilt‐corrected micrograph cross‐section of the manufactured surfaces, with the green dashed line showing the interface between the surface and, for imaging purposes, deposited tungsten. b) 3D topography reconstruction *S*(*x*,*y*,*z*) of the nanotopography by imaging surface cross‐sections with 5 nm spacing and 3.03 nm pixel size. c) Height map of the reconstructed topography with the origin in the bottom left corner, the *x*‐direction horizontal, and the *y*‐direction vertical. d) Cumulated distribution plot of the fraction of the surface area with radii smaller than |*R*|, *Q*, versus the absolute mean curvature radius |*R*|. The solid lines show both concave and convex parts of the surface. The dashed lines show only the concave, hence promoting part of the surface. The plot area from |*R*| < 10 nm to < 50 nm marks the range of one magnitude higher than the critical radius *r* ^*^. e) Spatial map of the variation of the geometric factor *f*(*θ* = 90°, *r* ^*^ = 2.13 nm, *R*) compared to *f*(*θ* = 90°, *r* ^*^ = 2.13 nm, *R* → ∞). The geometric factor influences the nucleation rate exponentially. Scale bar: a) 100 nm.

**Figure** **2**a shows the cross‐section of our nanoengineered surface, which we prepared for electron microscopy by filling the pits of the surface with tungsten (bright region) and milling the SiO_2_ surface (dark region) with a focused ion beam. To characterize the surface pattern, we first milled the surface in the *x*‐direction at *y* = 0, followed by taking a micrograph. We then determine the position of the tungsten‐SiO_2_ interface in the *z*‐direction (green dashed line). Sequential Δ*y* = 5 nm milling in the *x*‐direction and imaging steps are then done, allowing us to produce a three‐dimensional reconstruction of our surface, *S*(*x*,*y*,*z*), Figure 2b,c. Obtaining this reconstruction at higher resolution via atomic force microscopy (AFM) turns out to be impractical due to the convolution created by the probe tip geometries not reaching the 80 nm deep pits resulting in dead zones^[^ ^62^ ^]^ (Section S5, Supporting Information). Using the height map in Figure 2c, we can compute the mean surface curvature, *H*, at a point, *M*, by knowing $\hat{n}$ there, which is the unit normal to the surface. The radius of mean curvature is then *R* = 1/*H* (Section S6, Supporting Information). Figure 2d shows the cumulative distribution function of the fraction of the surface, *Q*, that possesses equal and smaller |*R*| at the nanoscale for the smooth, not including sub‐nanometer scale features introduced through the equal surface functionalization preparation of both surfaces (Section S7, Supporting Information) and nanoengineered case. We see for the nanoengineered surface that there is a substantial fraction of the surface with |*R*| < 50 nm, which is expected to be the upper bound^[^ ^31^ ^]^ for influencing the nucleation rate according to classical nucleation theory^[^ ^50^, ^51^ ^]^ (for smooth surfaces: Section S8, Supporting Information). Our nanoengineered surfaces consist of both convex and concave regions with |*R*| < 50 nm. The dashed line indicates that concave regions alone make up a substantial fraction of *Q* of the surface. As a consequence, convex regions make up a substantial portion of the surface, too.

To predict the likelihood of deposit nucleation on a given surface, previous work has analyzed individual features like bumps or pits with a single radius of curvature.^[^ ^52^, ^53^ ^]^ However, these analyses primarily concentrated on the first nucleation event rather than examining nucleation rates. What has not been explored is establishing the nucleation rate for each point *M* across the entire surface, considering varying curvatures. This approach allows one to quantify the effect of surface nanocurvature on the cumulative nucleation rate of a realistic surface. This is especially important when accessing the experimentally measured cumulative deposition of independently nucleating crystallites. Merely analyzing individual features is insufficient to model an overall surface nucleation rate. Hence, surface mapping of the collective features is necessary. Once we define the nucleation rate at each point on our smooth and nanoengineered surfaces, comparing their values provides mechanistic insight into the nucleation (Section S9, Supporting Information). First, following classical nucleation theory, we define the heterogeneous nucleation barrier at point *M* on the surface, *S*, as:

(1) $$\Delta G^{*}\left(M\right)=f\left(\theta ,\frac{R\left(M\right)}{r^{*}}\right)\Delta G_{H}^{*}$$

where $\Delta G_{H}^{*}$ is the homogeneous barrier to nucleation, *f* is the geometrical factor ≤ 1, by which the homogeneous nucleation barrier is reduced, which depends on the contact angle of the nucleating phase with the surface and the solution, *θ*, the critical radius of nucleation, *r* ^*^, and *R*. The nucleation rate can then be defined at each point over the surface as, $J\;=J_{\mathrm{kin}}\;\exp\left(-\frac{f\left(\theta ,R/r^{*}\right)\Delta G_{H}^{*}}{k_{B}T}\right)$, where *J*_kin_ is a kinetic prefactor, *k*_B_ is Boltzmann's constant, and *T* is temperature. Due to its exponential dependence, a small change in *f* causes a large change in *J*. Figure 2e shows a map on *S* of $\left[f-f(\theta ,\frac{R}{r^{*}}\to \infty )\right]/f\left(\theta ,\frac{R}{r^{*}}\to \infty \right)$ where *f* is determined by the properties of the nanoengineered surface (*θ* ≈90° and *r* ^*^ ≈2.13 nm; Section S9, Supporting Information). The nucleation rate increases exponentially for increases in *f* in concave nano‐pits. The collective effect of nano‐pits across the entire surface thermodynamically enhances the rate of nucleation. Guided by this theory, we proceed to experimentally explore these effects under various conditions to validate the feasibility of employing this approach for calcium carbonate formation.

**Figure 3:**
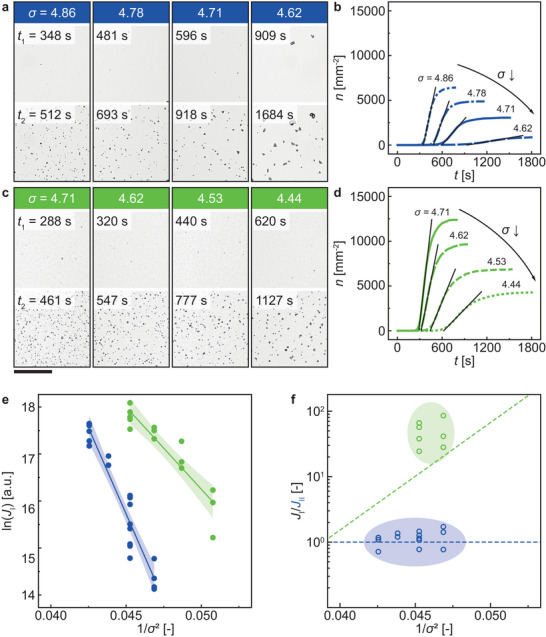
Nucleation rate evaluation at varying supersaturation conditions. Pairs of experimental images with increasing supersaturations for a) smooth and c) nanoengineered surfaces. The first image shows the experiment at the end of the induction time *t*_1_. The second image shows the experiment at the end of the nucleation dominant period with a linear increase of detected sites. b), d) Plot of the number of crystallites per area *n* versus time *t* for the a) smooth and c) nanoengineered surfaces. Line types indicate different supersaturations. (dot: *σ* = 4.44; short dash: *σ* = 4.53; long dash: *σ* = 4.62; solid: *σ* = 4.71; long dash dot: *σ* = 4.78; short dash dot: *σ* = 4.85). The linear nucleation dominant period is illustrated with a black solid line computed through linear regression. The slope of this line is the nucleation rate of that experiment. e) The nucleation rates of smooth (blue unfilled circle) and nanoengineered (green unfilled) surfaces in substrate position I (see Figure 1b) in n = 36 (smooth: n = 22, nanoengineered: n = 14 experiments). The solid lines are the least‐square linear regressions of the data points, with the surrounding shaded region being the 95% confidence interval for the slopes: Smooth 723.8 ± 53.2 (standard error); Nanoengineered 345.7 ± 40.5 (standard error). For the *y*‐intercepts: Smooth 48.2 ± 2.4 (standard error); Nanoengineered 33.5 ± 1.9 (standard error). f) The nucleation rate ratios in n = 22 experiments between substrate position I, shown in e), and substrate position II, which is always a smooth surface. The dashed lines are the ratios based on the linear regressions in e). Scale bar: 200 µm.

**Figure** **3**a,c show images of calcium carbonate nucleating on smooth and nanoengineered surfaces, respectively, under different supersaturation conditions. The top and bottom images denote the start and end of the period where the nucleating crystallites increase linearly with time, *t*_1_, and *t*_2_, respectively. Figure 3b,d show the corresponding plots of the crystallite number density, *n*, versus *t*. For decreasing values of *σ*, *t*_1_ and *t*_2_ – *t*_1_ increase. Additionally, *n*(*t = t*_2_) decreases with *σ*, implying increased mean distance between crystallites^[^ ^63^ ^]^ (Section S4, Supporting Information). Nanoengineered surfaces show calcium carbonate nucleation and growth at *σ* ≤ 4.53 within the experimental duration, possessing an analyzable linearly increasing nucleation curve. Smooth surfaces at those lower supersaturations show only a few crystallites that are not macroscopically shaped like calcite (Section S10, Supporting Information). Those differently shaped crystallites appear on all surfaces, likely induced by defects, at times before the linear increase starts at *t*_1_. Hence, for smooth surfaces at *σ* ≤ 4.53, *t*_1_ is larger than the experimental duration of our experiments. Figure 3e shows a plot of ln(*J*_I_) versus *σ* ^−2^ for all 36 experimental runs, where the “I” denotes the “bottom” substrate, which is either smooth or nanoengineered (see Figure 1b). Figure 3f shows a plot of *J*_I_/*J*_II_ versus *σ* ^−2^, where again, substrate “I” is either smooth or nanoengineered while “II” is the control surface, which is always smooth.

We see that when substrate “I” is a smooth surface, *J*_I_/*J*_II_ fluctuates around unity, indicating that neither substrate is enhancing nucleation, while when substrate “I” has a nanoengineered surface, *J*_I_/*J*_II_ the nanoengineered surface is enhancing nucleation, and even exceeds the expected enhancement ratio derived from the results in Figure 3e. This is because nucleation on substrate “II” starts only after a large number of crystallites exists on substrate “I” in the mixing zone, growing and thereby removing calcium and carbonate ions, decreasing the bulk supersaturation in the observation zone channel and thereby decreasing the expected nucleation rate (Section S11, Supporting Information). Substrate “II” still has a linear increasing part in its nucleation curve, showing that the reduced bulk supersaturation has an influence but not the counter‐surface in the observation zone channel. The linear increasing part of the nucleation curves on the substrate “I” occurs by design before the mixing zone in contact with substrate “I” can impact the bulk supersaturation. Since the flow regime in the observation zone is advection‐dominated, the two substrates do not influence each other, and the nucleation rates for substrate “I” in Figure 3e are independent at a known supersaturation. For both surfaces, ln(*J*_I_) has a linear dependence on *σ* ^−2^. Previous work has shown that nucleation behavior on smooth surfaces is predictable by classical nucleation theory,^[^ ^12^, ^14^ ^]^ but our nanoengineered surfaces also show classical dependency. We can analyze the slope, *B*, of the lines in Figure 3e to gain insight into the effect of supersaturation on the free energy barrier of nucleation as it is proportional to *B*. The slope of the linear regression, *B*, decreases from *B* ≈724 for the smooth surface to *B* ≈346 for the nanoengineered surface. We can also determine the effect of the surface properties on *J*_kin_, as the intercept is defined as ln(*J*_kin_). This value decreases from ln(*J*_kin_) ≈48 for smooth surfaces to ln(*J*_kin_) ≈34 for the nanoengineered surfaces.

From this, we see that the free energy barrier of nucleation for the nanoengineered surfaces is lower compared to the one on the smooth surfaces, while the kinetic barrier to nucleation is higher. The lower free energy barrier does not align with the phenomena of lattice strain inhibiting nucleation.^[^ ^18^, ^54^, ^58^ ^]^ The classical behavior of nanoengineered surfaces follows the mechanistic insight of thermodynamically enhancing the rate of nucleation through nanocurvature in the analysis of Figure 2. Since both surface types have the same surface chemistry, this could not have been predicted a priori without quantifying the surface nanotopography spatially. The analysis in Figure 2, using classical nucleation predictions incorporating curvature effects spatially, can interpret the experimental results in Figure 3e,f at different *σ*. The nanotopography resolution, here ≈15 nm^2^ pixel^−1^, plays a key role in the interpretation (Section S9, Supporting Information). The higher kinetic barrier to nucleation can be attributed to the collective confinement of the promoting nano‐pits, leading to kinetic restrictions compared to smooth surfaces. For equal *J*_kin_, nanoengineered surfaces enhance the nucleation rate for all *σ*. However, for reduced *J*_kin_, a threshold *σ* exists below which the nucleation rate on the nanoengineered surface is enhanced. The threshold is in a supersaturation regime for our surfaces, in which turbid solution containing ACC flows in our observation zone. The classical nucleation prediction of nanoengineered surfaces cannot be extrapolated into those regimes.

**Figure 4:**
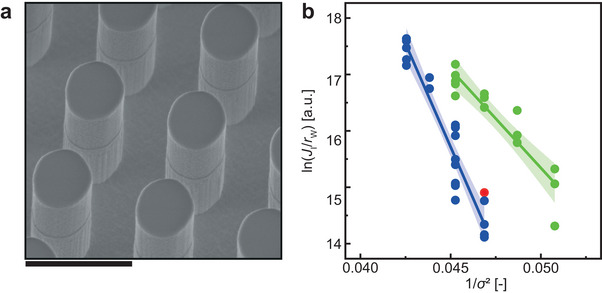
Microstructure impact on the nucleation rate. a) Tilted‐view micrograph of a micropillar array after fabrication before surface functionalization. Tilt angle: 30°. Scale bar: 2 µm. b) Nucleation rates, normalized by Wenzel surface roughness, *r*_W_, of smooth (blue), micropillar (red), and nanoengineered (green) surfaces in substrate position I (see Figure 3e) versus *σ* ^−2^. The linear regressions and the 95% confidence interval for the slopes are also shown for all the smooth and nanoengineered surfaces (smooth: *r*_W_ = 1.0, nanoengineered: *r*_W_ = 2.45, microstructure: *r*_W_ = 4.3). The red circle indicates the nucleation rate of the microstructured surface at *σ* = 4.62.

To demonstrate that the differences in nucleation rates are because of nanocurvature and not because of the increased surface area or changes in the formation of the SAM, we perform nucleation experiments on a microstructure, see **Figure** **4**a. This microstructure is characterized by the absence of features within the |*R*| < 50 nm range. Notably, it possesses a Wenzel roughness of *r*_W_ = 4.3, surpassing both the nanoengineered surface (*r*_W_ = 2.45) and the smooth surface (*r*_W_ = 1.0). Figure 4b illustrates the ln(*J*_I_ */r*_W_) versus *σ* ^−2^ plot previously presented in Figure 3e without correcting for *r*_W_. This adjustment resulted in an absolute reduction of 0.90 for the *y*‐intercept specific to the nanoengineered surface, indicating an even higher kinetic barrier to nucleation. The slope, which contains thermodynamic information, stays unchanged. This unchanged slope shows that the increased surface area is not a major contributor to the nucleation enhancement. Additionally, we marked the nucleation rate of the microtopography surface at *σ* = 4.62 using a red circle. This surface underwent identical surface preparation procedures to form the SAM. The microstructure nucleation rate, adjacent to the linear regression line of the smooth surface, strongly indicates that the enhancement in nucleation rate is driven by the nanocurvature effect rather than by increased roughness due to surface fabrication,^[^ ^32^, ^64^ ^]^ dominating active defects of even smaller size^[^ ^65^, ^66^, ^67^, ^68^, ^69^ ^]^ (Section S12, Supporting Information) or SAM formation differences. This conclusion underscores the significance of nanocurvature in influencing nucleation rates, providing valuable insights into the surface‐induced nucleation mechanism.

**Figure 5:**
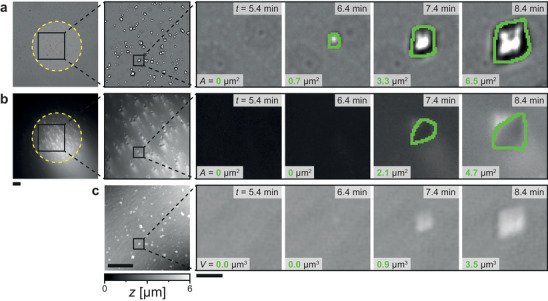
Simultaneous acquisition of single crystal growth. Images sequences at *σ* = 4.53 of a single calcite crystal nucleating and growing on a nanoengineered surface at substrate position I (see Figure 1b) for a) brightfield imaging, b) TIRF microscopy imaging, and c) digital holographic microscopy (DHM) imaging. The greyscale values of the brightfield images depend on how much light reaches the camera sensor, the grey values of the TIRF images are emitted light of an added fluorophore (Fluorescein sodium salt), and the greyscale values of the DHM images are height values after phase reconstruction of the acquired hologram. Each image shows the detected area *A* and computed volume *V*. The green outline of the crystals in a) is calculated using instance segmentation. The usage of one fluorophore molecule per 100 calcium ions impacts the nucleation behavior of calcium carbonate. Using the fluorophore and the TIRF excitation laser (yellow dashed circle indicates spot location) induces local laser‐induced nucleation in the spot before it occurs outside the spot, hence additionally impacting the nucleation behavior, likely through local heat transfer from fluorophore to its environment, increasing the supersaturation around excited fluorophore molecules. The difference in refractive indices between crystal and replaced water alters the light path at a crystallite site from reflection to refraction, introducing background illumination. Object detection detects the crystal in the brightfield image before it is detected in the TIRF image. Scale bar 1st and 2nd column: 20 µm; magnified sequences 3rd–6th column: 2 µm.

To observe crystal growth on our surfaces, **Figure** **5** shows a simultaneous acquisition of optical brightfield microscopy in Figure 5a, TIRF microscopy in Figure 5b, and DHM in Figure 5c of a nanoengineered surface at *σ *= 4.53. Brightfield microscopy reveals the crystallite's projected area, while TIRF isolates the projected contact area, which is not the true contact area, of calcium carbonate sites by exploiting the exponentially decaying evanescent wave to excite fluorophore molecules near the interface in the solution (Section S13, Supporting Information). DHM phase reconstruction offers volume information of each crystallite. This method enables quantification of surface area and volume evolution, revealing growth rates along the surface interface and into the bulk over time. Compared to smooth and nanoengineered surfaces, the latter exhibit lower area and volume growth rates. The used fluorophore molecules impact nucleation behavior, increasing rates and site density for nanoengineered surfaces while increasing rates and decreasing site density for smooth surfaces. The excited fluorophore in the laser reflection spot enhances local, laser‐induced nucleation, creating heterogeneous conditions across surfaces and introducing background illumination through refraction at the surface crystallite interface, distorting extracted area information. To evaluate growth rates with known nucleation behavior, we proceed with fluorophore‐free solutions without using TIRF, building on Figure 3’s nucleation experiment results.

**Figure 6:**
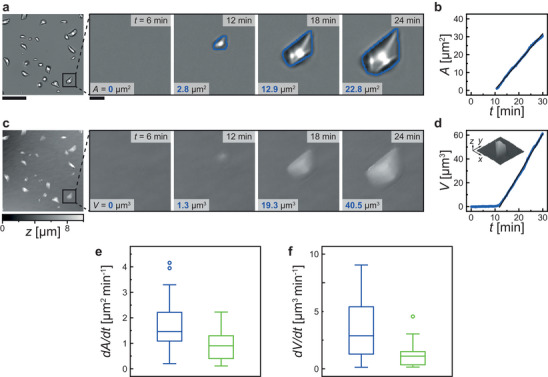
Single crystal volume and area growth analysis through non‐invasive optical measurements. Simultaneous image sequences at *σ* = 4.71 of a single calcite crystal nucleating and growing on a smooth surface at substrate position I (see Figure 1b) for a) brightfield imaging and c) digital holographic microscopy (DHM) imaging. The greyscale values of the brightfield images depend on how much light reaches the camera sensor. The greyscale values of the DHM images are height values after phase reconstruction of the acquired hologram. The detected area *A* and computed volume *V* in each image is shown. The blue outline of the crystals in a) is calculated using instance segmentation. The evolution of b) the area and d) the volume versus time *t* of the single crystal is plotted in blue. The black solid lines are the linear regressions. The inset shows a 3D plot of the crystal after 24 min. The evaluated area growth rate e) and volume growth rate f) are plotted as box plots for smooth (blue) and nanoengineered (green) surfaces for *σ* = 4.71. The boxes extend from the lower to upper quartile values, with a line at the median, whiskers showing the range of the data, and outliers are those past the end of the whiskers. Smooth: n = 57 single crystals; Nanoengineered: n = 45 single crystals. T‐test: e) P‐value = 2 × 10^−5^; f) P‐value = 7 × 10^−8^. Scale bar: 20 µm; magnified inset: 2 µm.

**Figure** **6**a–d shows the behavior of a single crystallite on the smooth surface without fluorophore in the aqueous solution (for the nanoengineered substate: Section S14, Supporting Information). Brightfield images and instance segmentation were used to obtain the projected area *A* of each crystallite, plotted versus *t* in Figure 6b. The detected location of the single crystallite was used to evaluate the DHM phase reconstruction, and the crystallite's volume *V* was plotted versus *t* in Figure 6d (Movie S3, Supporting Information). Regardless of the surface type, the only calcium carbonate polymorph observed is calcite. While this is known for smooth carboxyl‐terminated surfaces,^[^ ^12^, ^14^, ^23^ ^]^ it is a new insight for nanoengineered surfaces. In our analysis in Figure 2 with the results in Figure 3, the nucleation probability is highest in the pits within the confined volume of the nanotopography. Previous work on confined volumes (not within nanotopographies) with sizes ≈50 nm showed a change in the nucleation pathway toward aragonite^[^ ^22^ ^]^ or kinetically stabilized ACC.^[^ ^20^, ^21^ ^]^ Calcite crystals, emerging macroscopically from nano‐pits, despite conditions suggesting a relation to other polymorphs, and given the nanocurvature mismatch with the calcite crystal lattice, challenge intuitive expectations. While optical microscopy provides this insight, it is unable to measure initially present ACC layers reported for phlogopite micas.^[^ ^11^ ^]^ Using modeling approaches, like molecular dynamics, on feature sizes of our study could provide a further understanding of whether a metastable calcium carbonate phase is initially present^[^ ^11^ ^]^ on nanocurvatures and whether an amorphous layer exists, like for ice nucleation,^[^ ^52^ ^]^ to prevent inhibition effects of curvature as seen for aspirin.^[^ ^54^ ^]^ Such atomistic perspectives might be necessary to look into in the future. In Figure 6b, the plot shows the growth behavior of *A*, increasing linearly with time once the crystallite is detected. Figure 6d shows that *V* follows the same linear growth. Before the crystal is detected, the *V* already increases, gradually building up to the linear growth phase. This buildup suggests varying growth rates during the early stages of crystallite development.

Figure 6e,f displays the growth rates, *dA*/*dt*, and *dV*/*dt*, for multiple single crystallites at *σ *= 4.71 for smooth and nanoengineered surfaces. Both datasets show statistically significantly higher rates on the smooth surface. Operating under flow conditions, preventing diffusion limitation (*Pe* > 10^3^), nucleation events become dependent on each other over time, exemplifying that growth rates are dependent on the site density and, therefore, crystallite spacing. Thus, the smooth surface exhibits a 96% larger crystallite spacing at *σ *= 4.71. On nanoengineered surfaces at different conditions (Figure S8e,f, Supporting Information), the lower *σ* shows a 35% larger spacing and larger growth rates. Considering site densities (smooth: ≈3000 mm^−2^; nanoengineered: ≈12500 mm^−2^ at *σ *= 4.71) and mean volume growth rates (smooth: ≈3.6 µm^3^ min^−1^; nanoengineered: ≈1.1 µm^3^ min^−1^ at *σ *= 4.71), the total calcium carbonate deposition rate across the entire nanoengineered surfaces is ≈18% larger. On the surfaces at different conditions (Section S14, Supporting Information), the total deposition rate is higher at higher supersaturations, with higher site densities at larger supersaturations dominating over the reduced single crystallite volume growth. The ratios $\frac{d\sqrt[{3}]{V}}{dt}/\frac{d\sqrt[{2}]{A}}{dt}$ at different conditions and different surfaces are not statistically significant. Hence, the nanotopography does not influence the growth along the surface beyond altering the crystallite spacing.

## 3 Conclusion

We have elucidated the effects of surface nanocurvature and supersaturation on calcium carbonate nucleation and growth. Through optical measurements, spatial reconstruction of nanotopographies, and a cumulative model with spatially dependent nucleation rates, we have established that nanoengineered surfaces substantially reduce the thermodynamic nucleation barrier, which follows predictions of classical nucleation theory, extending the classical description of smooth surfaces to nanoengineered ones. Our nanoengineered surfaces experienced order of magnitudes higher nucleation rates, showing that nanotopography can promote nucleation although increasing the kinetic barrier. This shows that nano‐pits and their confinement are the key properties to control calcium carbonate formation. On nanoengineering and smooth surfaces, calcite is the calcium carbonate polymorph that is observed to grow macroscopically. Hence, confined nano‐pits do not alter the nucleation pathway to a different macroscopically grown polymorph. Further, we investigated the growth of crystallites determining the area and volume evolution, showing lower growth rates on nanoengineered surfaces related only to the crystallite spacing and not to an influence of nanocurvature. These findings on the influence of surface nanocurvature on mineral scale nucleation and growth could have profound implications on the development of interfaces, exploiting the thermodynamic enhancements or the kinetic restrictions. The design of heat transfer or membrane surfaces, preventing crystallization fouling, must incorporate these findings and avoid kinetically available concave nanocurvature to develop intrinsically scalephobic surfaces.

## 4 Experimental Section

### Nanotopography Fabrication

The nanotopography was fabricated on precision cover glasses (Thorlabs Inc., CG15CH). To clean the cover glasses, they were first sonicated in acetone, 2‐Propanol, and deionized water (Direct‐Q 3 System) for 5 min each, followed by drying in nitrogen. Subsequently, the cover glasses were treated with oxygen plasma (200 W, 5 min) to remove all remaining organics. With plasma‐enhanced chemical vapor deposition (Oxford Instruments PlasmaPro 100 PECVD), a 120 nm thick layer of SiO_2_ was deposited to eliminate the influence of the underlying borosilicate glass. Poly(styrene‐co‐methyl methacrylate), (α‐Hydroxyl‐ω‐Tempo)‐terminated (Polymer Source. Inc., P9085‐SMMAranOHT) was mixed at 1 wt.% in toluene and spin‐coated (1500 rpm, 30 s, 2000 rpm s^−1^) on the oxygen plasma activated surfaces, followed by thermal annealing at 200 °C for 4 h at <10 mbar and subsequent rinsing with toluene to form a brush layer. Poly(styrene)‐b‐poly(methyl methacrylate) (Polymer Source. Inc., P6402‐SMMA) was mixed at 1 wt.% in toluene and spin‐coated (3000 rpm, 30 s, 2000 rpm s^−1^) on the brush layer, followed by thermal annealing at 200 °C for 12 h at <10 mbar to form a self‐assembled block copolymer layer. Thermal atomic layer deposition (ALD) was used for block‐selective infiltration of the block copolymer structures. Trimethylaluminum (TMA) (Al(CH_3_)_3_, 97% purity from STREM) and distilled water were employed in a customized ALD reactor running at 85 °C, 2 × 10^−2^ mbar background pressure, and with argon (Ar) as purge and carrier gas. The glass cover slides were vertically placed ≈3 mm behind each other in the reactor. The protocol used a sequence of three repeated TMA half cycles followed by three repeated H_2_O half cycles, which were repeated four times in total. Each half cycle was composed of 0.4 s precursor opening at 15 sccm Ar (exhaust valve closed), 30 s of exposure of the samples in the reactor (exhaust valve closed), and a 180 s purge (exhaust valve open) at 100 sccm Ar. Both precursors were kept at room temperature. Subsequent oxygen plasma treatment (200 W, 5 min) removed most of the organic material, and left an aluminum oxide mask. This mask was transferred to the SiO_2_ layer by anisotropic inductively coupled plasma (ICP) etching (Oxford Instruments PlasmaPro 100 Cobra) using a fluoroform CHF_3_ (30 sccm) and oxygen (10 sccm) plasma mixture at room temperature and 15 mTorr, with 700 W ICP power and 40 W radio frequency (RF) power for 3 min (etching rate 30 nm min^−1^). Both surface types are macroscopically smooth with root‐mean‐square roughness < 25 nm. Previous work on similar nanotopography interfaces influenced multiple phenomena, from enhanced antireflection,^[^ ^70^ ^]^ enhanced transmittance,^[^ ^71^ ^]^ and robust superhydrophobicity^[^ ^72^ ^]^ to anti‐fogging.^[^ ^73^ ^]^

### Microstructure Fabrication

The microstructure was fabricated on a 4‐inch silicon wafer. To clean the wafer, it was first treated with oxygen plasma 600 W for 5 min to remove any remaining organics. Then, the silicon wafer was pretreated with HMDS (hexamethyldisilazane) at 110 °C for 1 min to promote photoresist adhesion on the inorganic wafer. A 0.5 µm thick layer of diluted AZ nLOF 2020 photoresist was spin‐coated at 3000 rpm for 40 s with a ramp of 2000 rpms^−1^. The wafer was then baked at 110 °C for 1 min. The photoresist was exposed using a mask aligner (MA6) for 0.8 s to transfer the pattern. Then, the wafer was developed using the developer AZ 726 MIF. Next, we etched the wafer by deep reactive ion etching (Alcatel AMS 200SE I‐Speeder) using a SF_6_ plasma. Post‐etching, oxygen plasma 600 W, 5 min was used to remove the remaining photoresist from the silicon wafer, followed by drying under nitrogen. With plasma‐enhanced chemical vapor deposition (Oxford Instruments PlasmaPro 100 PECVD), a 200 nm thick layer of SiO_2_ was deposited to eliminate sharp corners below radii of 200 nm.

### Surface Functionalization

To functionalize the smooth and nanoengineered glass coverslips, they were passivated with 2 nm SiO_2_ using ALD (Oxford Instruments FlexAL ALD) and oxygen plasma treatment for activation. The activated surfaces were treated for 48 h by direct evaporation of (3‐Mercaptopropyl)trimethoxysilane (Sigma–Aldrich, 95%) at 5–10 mbar. Subsequently, 8 nm gold was evaporated (Evatec BAK501 LL) to form a layer beyond the percolation threshold due to the pre‐treatment.^[^ ^74^ ^]^ The surfaces are storable, and SAMs were deposited by submerging the surfaces for 24–30 h in a 2 mm solution of 16‐Mercaptohexadecanoic acid (Sigma–Aldrich, 90%) of 95% ethanol (Supelco.) and 5% acetic acid (Sigma–Aldrich, ≥ 99%). The surfaces were rinsed with the solvent and dried with nitrogen directly before assembling the surface in the microfluidic chip. The equal chemical functionalization of both surface types with a thiol‐based self‐assembling monolayer ensures chemical defect comparability^[^ ^60^ ^]^ and isolates the nanotopography as the only influence on the thermodynamic nucleation barrier.

### Crystallization Measurements

Experimental solutions were prepared directly before each experimental run, diluting 100 mm solutions of calcium chloride dihydrate (Supelco.) and sodium carbonate (VWR, 99.5–100.5%), which were prepared on the same day. The solutions were individually transferred to polypropylene/‐ethylene syringes (Braun Injekt) and with PTFE/Tygon (Darwin Microfluidics), silicone (microfluidic ChipShop) tubing, and bubble traps (Darwin Microfluidics) connected to the microfluidic chip (Figure S2, Supporting Information; Silicon rubber: Steinbach AG; PET sheets: Bleher Folientechnik GmbH). A syringe pump (Havard Apparatus, 33 Syringe Pump Dual) continuously flowed the solutions at $\dot{V}$ = 250 µL min^−1^ per syringe to ensure homogeneous supersaturation conditions at *Re* < 10 in the region of interest. The microfluidic geometries in the observation zones are too large to confine calcium carbonate nucleation^[^ ^19^, ^20^, ^21^, ^22^ ^]^ and prevent supersaturation changes for the nucleation rate analysis through deposits in the mixing zone^[^ ^75^ ^]^ (Section S3, Supporting Information). The experiments were conducted at supersaturations of *σ* = 4.44–4.86 (Geochemist's Workbench,^[^ ^10^, ^14^ ^]^ *thermos_phreeqc.tdat*), at pH = 10.5 ± 0.1, and T = 22.5 ± 0.5 °C (Section S2, Supporting Information). The crystallization was monitored by optical and digital holographic microscopy (Nikon Ti2‐E, Lyncée Tec DHM) at 0.5 FPS for each substrate position. The height analysis of the reconstructed holograms was validated by ex situ profilometer measurements (DektakXT, stylus radius: 5 µm, stylus force: 3 mg), collecting 3D maps in the observation zone (Section S15, Supporting Information). The crystallites were detected once they had grown large enough. Hence, the nucleation event occurred before the first detection. A motorized stage moved the objective up and down to image substrate positions I and II (see Figure 1b).

### Image Characterization

The brightfield images for nucleation rate determinations were segmented with Fiji,^[^ ^76^ ^]^ using non‐local means denoising,^[^ ^77^ ^]^ background subtraction, edge detection, and binary thresholding. The object count was computed using the OpenCV Python module. The micrograph cross‐section images to reconstruct the topography of the nanoengineered surface were aligned with a template matching plugin in Fiji, followed by segmentation with the same algorithms as the brightfield images for nucleation rate determinations. The projected area of crystallites in brightfield images for single crystal observation was analyzed with a trained instance segmentation algorithm using Detectron2^[^ ^78^ ^]^ and tracked using TrackMate 7.^[^ ^79^ ^]^ A total loss of 0.06624 was reached after 4.8 × 10^5^ training steps with a training dataset labeled by us. The bounding box mean average precision is 71.5 and the segmentation mean average precision is 69.2.

### Atomic Force Microscopy

AFM scans were collected on an Asylum Research AFM (MFP‐3D Origin, Oxford instruments) in air tapping mode with PPP‐NCH probes (NANOSENSORS).

### Focused Ion Beam Scanning Electron Microscopy

Micrographs were collected in a TFS Helios 5 UX (Thermo Fisher Scientific). If needed, the samples were coated with 10 nm Tungsten in a metal sputter coater (Safematic CCU‐010) for tilted and top‐view images.

### Statistical Analysis

Data were statistically analyzed without pre‐processing using the Python module SciPy.^[^ ^80^ ^]^ The sample size, n, for each analysis, is given in the corresponding figure caption. The 95% confidence intervals of the population mean for the least‐square linear regressions were calculated using the scipy.stats.t.ppf function and the values are presented as mean ± standard error of the mean. The T‐test of two independent samples was calculated using the scipy.stats.ttest_ind function and was used to test for the null hypothesis that the samples have identical average values. A P‐value below 0.05 was considered to be significantly different.

## Conflict of Interest

The authors declare no conflict of interest.

## Supporting information

Supporting Information

Supplemental Movie 1

Supplemental Movie 2

Supplemental Movie 3

## Data Availability

The data that support the findings of this study are openly available in the Eidgenössche Technische Hochschule Research Collection at https://doi.org/10.3929/ethz‐b‐000648538.
