# Supplementary material for: Nanostructured Surfaces Enhance Nucleation Rate of Calcium Carbonate
Source: Small. 2024 Aug 20;20(47):2402690. doi: 10.1002/smll.202402690 (PMC11579968; doi:10.1002/smll.202402690)
Supplement: Supplementary file 1 — Supporting Information [file SMLL-20-2402690-s002.docx]

Supporting Information

Nanostructured Surfaces Enhance Nucleation Rate of Calcium Carbonate

Tobias Armstrong, Julian Schmid, Janne-Petteri Niemelä, Ivo Utke, Thomas M. Schutzius^*^

This PDF file includes:

Supporting text Sections S1 to S15

Figures S1 to S18

Table S1

Legends for Movies S1 to S3

SI References

**S1: Step-by-step nanoengineered surface fabrication**


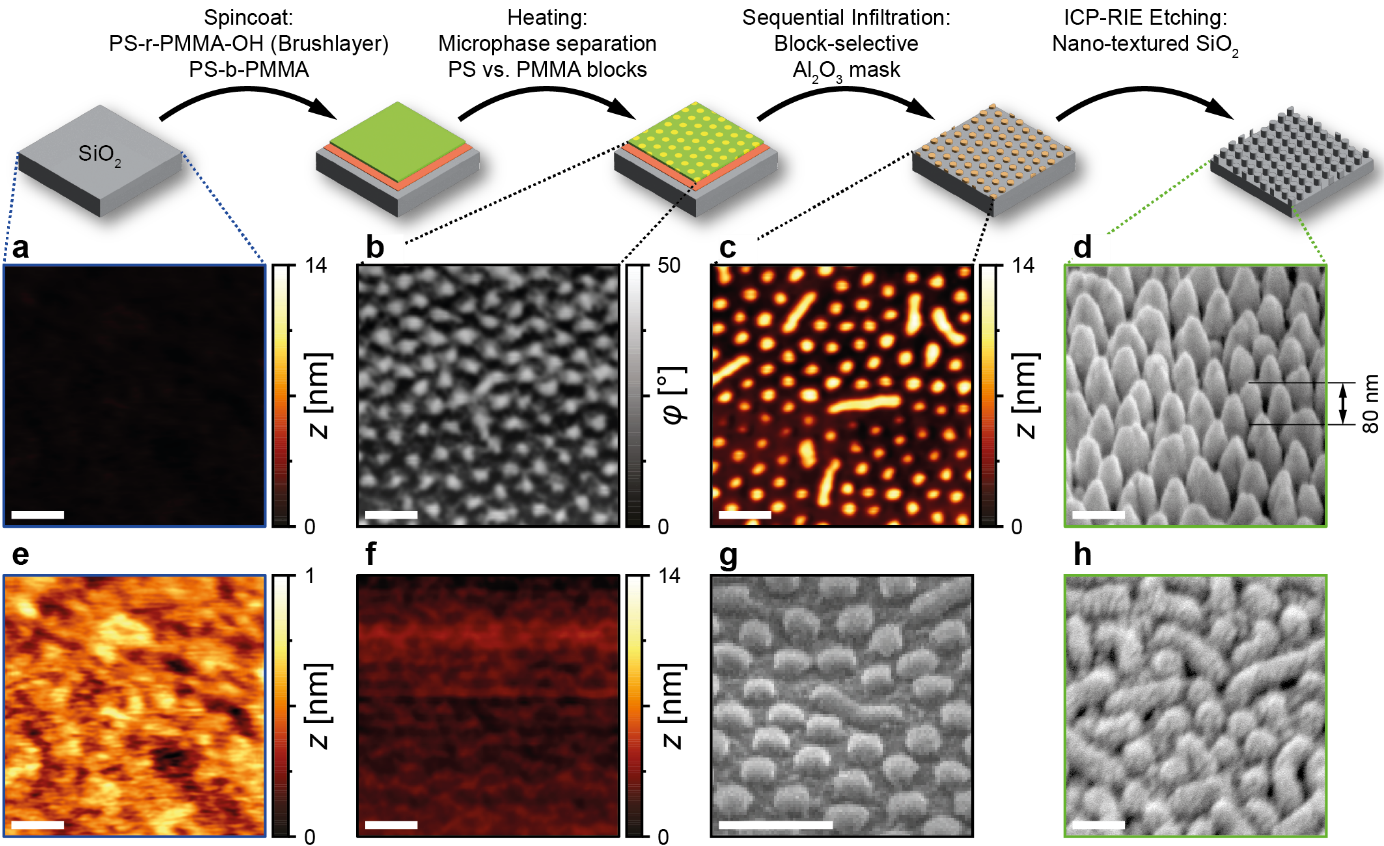


**Figure S1. Manufacturing of nanoengineered surfaces on precision glass coverslips**. a) Atomic force microscopy (AFM) height scan of precision glass coverslips passivated with 2 nm SiO_2_ using atomic layer deposition (ALD). b) AFM phase scan after phase-separation of block copolymer PS-b-PMMA. For successful phase separation, heating of the block copolymer layer on a brush layer is required here: PS-r-PMMA-OH. c) AFM height scan of the Al_2_O_3_ hard mask after selectively infiltrating Al2O3 into the PMMA block domains and subsequent removal of all organic content with oxygen plasma. d) Tilted (52°) micrograph after inductively coupled plasma (ICP) etching, transferring the hard mask pattern into the SiO_2_ surface. The tilt of the micrograph was corrected; hence the measured height is the real height based on the scale bar. e) AFM height scan in a) with changed color bar boundaries. f) AFM height scan of the phase scan in b). g) Tilted (25°) micrograph of the Al_2_O_3_ hard mask with tilt correction. h) Tilted (30°) micrograph of the nanoengineered surface after surface functionalization (see Methods) without tilt correction. Scale bars: 100 nm.

**S2: Supersaturation computation**

**Table S1.** **Computed supersaturations of used solution composition after mixing.** The inputs needed to compute the supersaturation are solution concentrations, temperature, and pH value. The computation was conducted using Geochemist’s Workbench and the thermodynamic dataset “thermos_phreeqc.tdat”.

| Ca^2+^  [mmol/L] | Cl^-^  [mmol/L] | Na+  [mmol/L] | CO32-  [mmol/L] | *T*  [°C] | *pH*  [-] | *σ*  [-] |
| --- | --- | --- | --- | --- | --- | --- |
| 1.9 | 3.8 | 3.8 | 1.9 | 22.5 | 10.5 | 4.855 |
| 1.8 | 3.6 | 3.6 | 1.8 | 22.5 | 10.5 | 4.783 |
| 1.7 | 3.4 | 3.4 | 1.7 | 22.5 | 10.5 | 4.705 |
| 1.6 | 3.2 | 3.2 | 1.6 | 22.5 | 10.5 | 4.623 |
| 1.5 | 3.0 | 3.0 | 1.5 | 22.5 | 10.5 | 4.534 |
| 1.4 | 2.8 | 2.8 | 1.4 | 22.5 | 10.5 | 4.439 |

**S3: Microfluidic stack design**


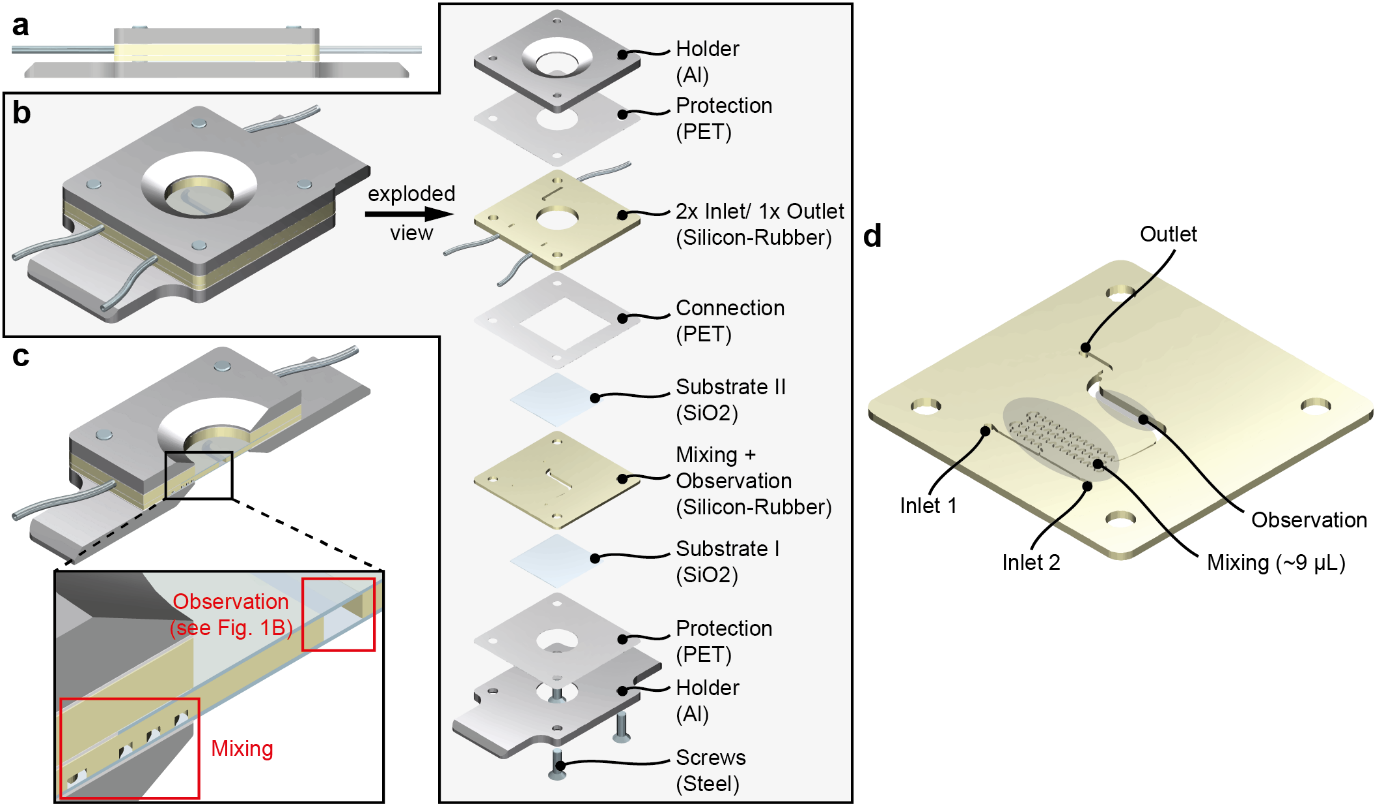


**Figure S2. Microfluidic stack with mixing and observation zone of two optical accessible substrates.** Reusable microfluidic stacked chip design for testing two substrates in one experimental run. a) Rendered side-view of schematic illustration in Figure 1a. b) Isometric rendering with exploded view showing every stack layers function and material: Aluminum holder with steel screws to clamp all layers; PET protection and connection layer to properly seal the stack and ensure fluid contact with only the PET, the silicon rubber, and the substrates; Silicon-Rubber sheet to connect tubing for inlet and outlet; Substrate I and II to study crystallization; Silicon-Rubber sheet to mix (in contact with Substrate I) and observe the solution. c) Cut isometric view with zoom to illustrate the mixing and observation zones in the clamped stack. d) Flipped Silicon-Rubber sheet with marked in-/outlets, the mixing zone (≈ 9 µL), and the observation zone.

**S3.1: Full mixing validation**

To characterize whether or not the aqueous solutions were fully mixed upon entering the observation zone, we used a fluorophore (fluorescein sodium salt) with a lower diffusion coefficient (0.42 · 10^-5^ cm^2^/s)^[1]^ due to its larger molecule size than calcium or carbonate ions (0.79 · 10^-5^ cm^2^/s and 0.92 · 10^-5^ cm^2^/s respectively)^[2]^. **Figure S3** shows the mixing behavior by imaging the emission of the excited fluorophore in the observation zone. No mixing is illustrated in Figure S3a, while fluorophore in both inlet inlets is illustrated in Figure S3c. Figure S3b shows the flow conditions of our crystallization experiments with fluorophores added to one inlet. The resulting emission intensities show that it is similar to Figure S3c and we have full mixing conditions. Given that the fluorophore for the experimental conditions is fully mixed, it can be inferred that calcium and carbonate ions will also fully mix under these conditions. Hence, this results in homogeneous supersaturation in the observation zone.


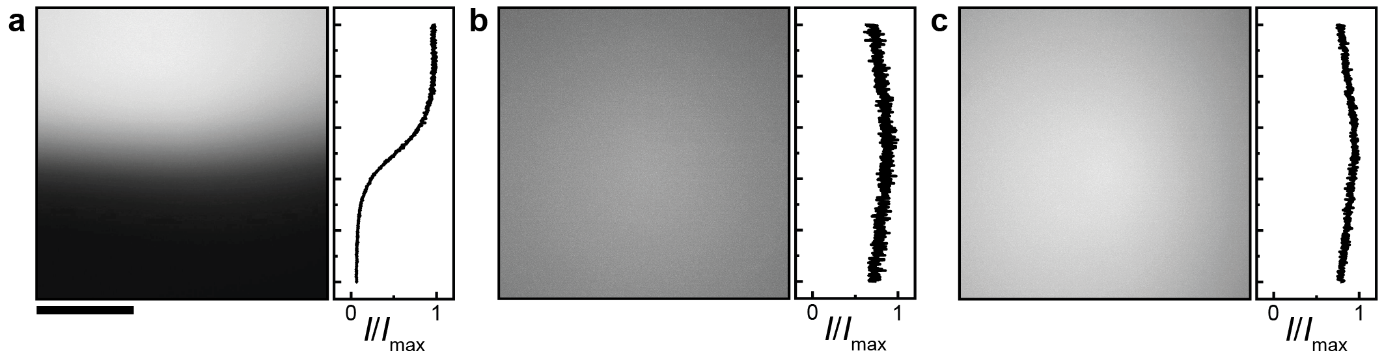


**Figure S3. Mixing validation of microfluidic chip.** Fluorescent images of the observation zone Figure S2d at $\dot{V}$ = 500 μL/min in the microfluidic chip. Aqueous solutions were used with fluorescein sodium salt (Sigma Aldrich) as a fluorescent tracer with a diffusivity coefficient larger than calcium and carbonate ions. The normalized intensities I/I_max_ are plotted next to the corresponding image for an observation zone: a) without a prior mixing zone and one of two inlets containing the tracer; b) with the mixing zone in Figure S2d and one of two inlets containing the tracer; c) without a prior mixing zone and both inlets containing the tracer. Successful mixing of molecules of lower diffusion coefficients guarantees successful mixing of ions of higher diffusion coefficients. Scale bar: 200 µm.

**S3.2: Consistent advection-driven supply of the chosen *σ***

**Figure S4** shows the theoretical flow profile in the channel solving the Navier-Stokes equation for laminar flow and the contour line of Péclet number, *Pe* = *d v(d*,*w)/D* = 1, where *D* is the diffusion coefficient of calcium ions^[2]^. This shows that a 3.6 µm thick layer at the surface is diffusion-dominated while the bulk in the channel is advection-dominated. This consistent advection-driven supply of the chosen supersaturation in the entire, uniformly nucleating observation zone channel (see **Figure S5**) lets the bulk supersaturation seen by the top surface be independent of nucleation on the bottom surface in the observation zone. The rectangular channel prevents recirculation zones. The microfluidic approach ensures a large surface area to volume ratio in the system, preventing bulk impurities from affecting the measurements^[3]^. The dominant advective mass transport prevents the influence of depletion on the nucleation rate dataset in Figure 3e^[4]^. The microscope objective is centered between the channel walls. The position along the channel was not relevant and mostly at half the channel length for operational convenience.


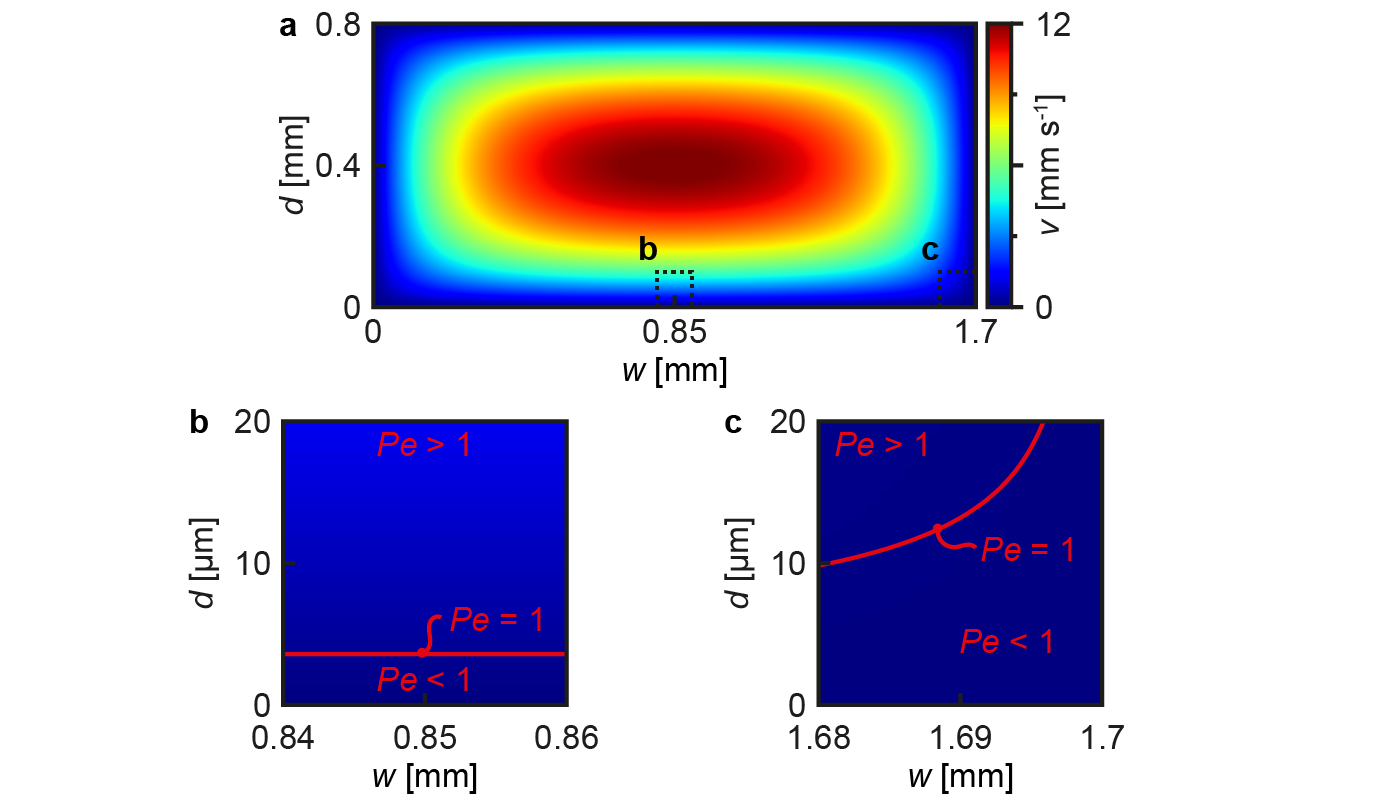


**Figure S4. Flow and mass transport conditions in the observation zone.** a) Cross-sectional view of the flow velocity magnitude within our test section; w is the width of the channel, and d is the height of our channel (see Figure S2d). Magnified views of the velocity magnitude, v, near the substrate in the b) middle of the channel and c) near the edge. In b) and c), we note the regions where there is advection-dominated mass transport, Pe = d v(d,w)/D > 1, and diffusion-dominated mass transport, Pe < 1, where D is the diffusivity of the calcium ions in water.


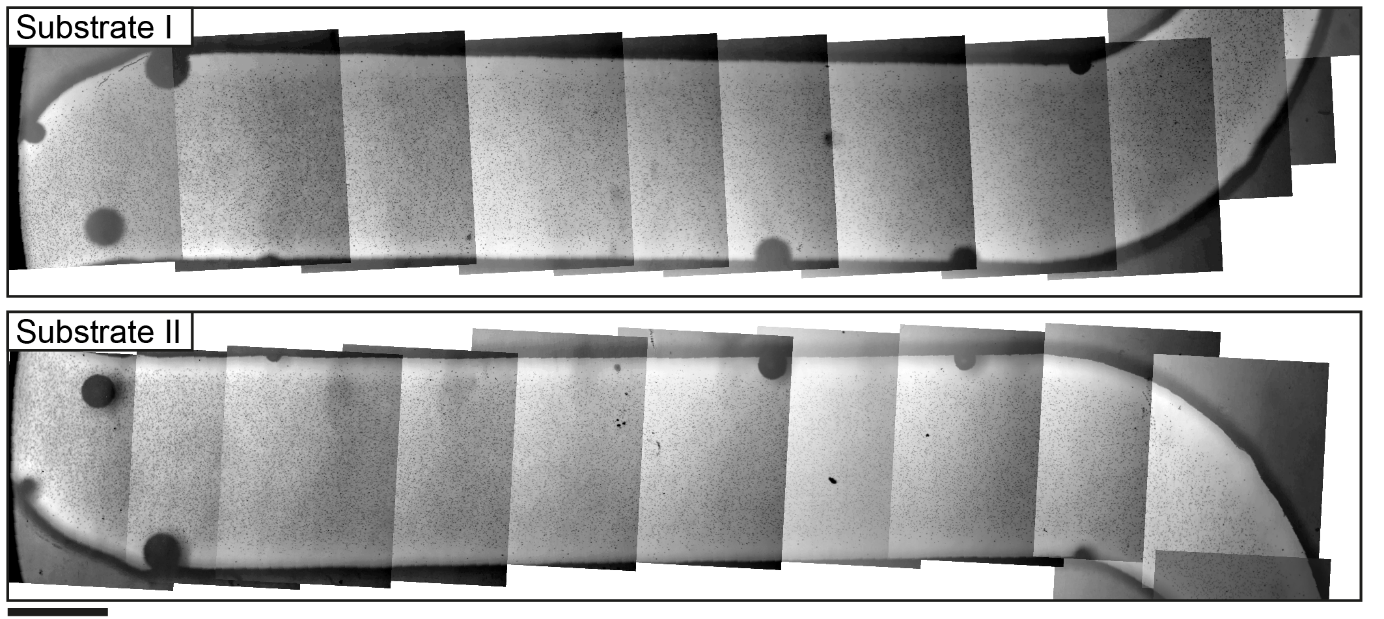


**Figure S5. Crystallization along the channel.** Stitched images of the observation zone channel in the microfluidic stack. The images have been taken after 30 min of calcium carbonate nucleation at σ = 4.71 for smooth surfaces in both substrate positions (I: bottom substrate, II: top substrate). There are numerous small crystallites (seen as small black objects) along the entire observation zone on both substrates. The dark regions on the sides of the observation zone are the channel walls. Scale bar: 1 mm.

**S4: Local crystallite effects**

**Figure S6** schematically shows that a forming site forms a local diffusion zone. This zone forms because diffusion dominates near the surface^[5]^ in a thin boundary layer (3.6 µm thick for our experimental conditions). The solution at the crystallite interface is saturated, while the advection-dominated bulk supplies the boundary layer with the set supersaturation, creating a supersaturation gradient. Due to this local diffusion around an existing zone, the supersaturation at the solution-surface interface in that zone is gradually lower, decreasing the nucleation rate accordingly. In our images, the affected area is negligible for small numbers of sites since it is significantly smaller than the overall overserved area. The affected area is no longer negligible for large numbers of sites, which means that further nucleation events in the overall image are no longer independent^[6]^. This ends the linearly increasing part of the nucleation rate curve, whose slope is the independent nucleation rate. The duration *t*_2_-*t*_1_ of this slope increases with decreasing supersaturation since the nucleation rate decreases with decreasing supersaturation, and it takes longer to fill the observed area with nucleating sites until further nucleation events are no longer independent. At increased supersaturation, the crystallite’s diffusion zone has a larger supersaturation gradient, and at the surface, a smaller area around the crystallite exists in which the nucleation rate decreases to an extent that no nucleation occurs anymore within the experimental duration. A larger area around a crystallite exists at increased supersaturations in which the nucleation rate is still substantially large enough to nucleate a new site within the experimental duration. Therefore, larger site densities, *n*, are observed at increased supersaturations, and lower *n* are observed at decreased supersaturations.


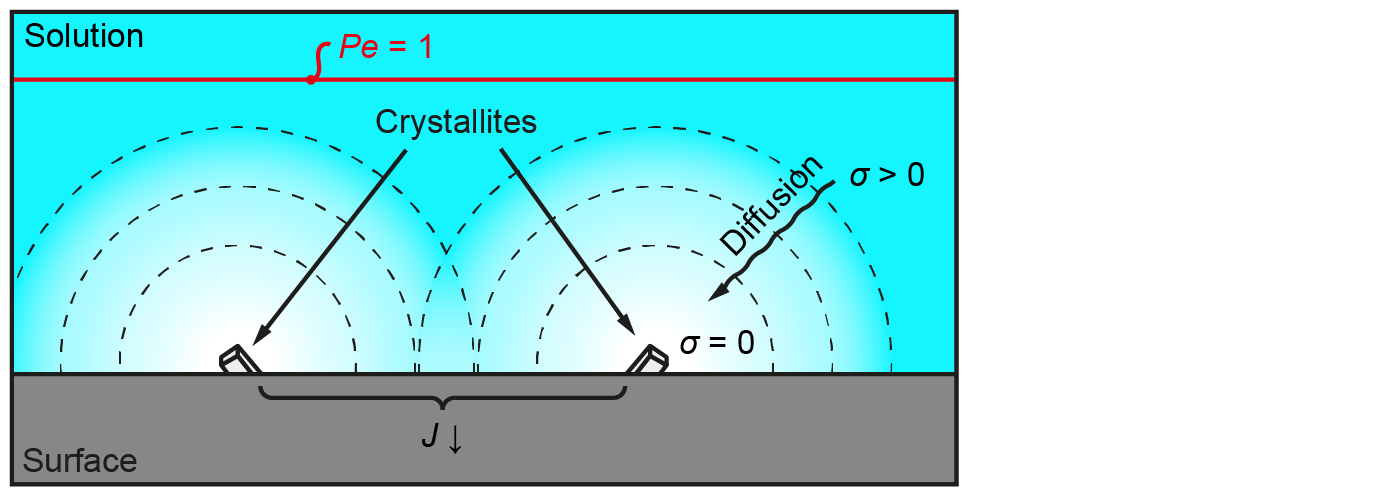


**Figure S6. Schematic of crystallite effects on local supersaturation.** The advective regime in the bulk maintains constant supersaturation. The growing crystal in a thin diffusion-dominated boundary layer creates a supersaturation gradient in its vicinity. This results in a reduced nucleation rate J in the vicinity of the already existing nucleation site.

**S5: Limits of AFM characterization for nanoengineered surfaces**


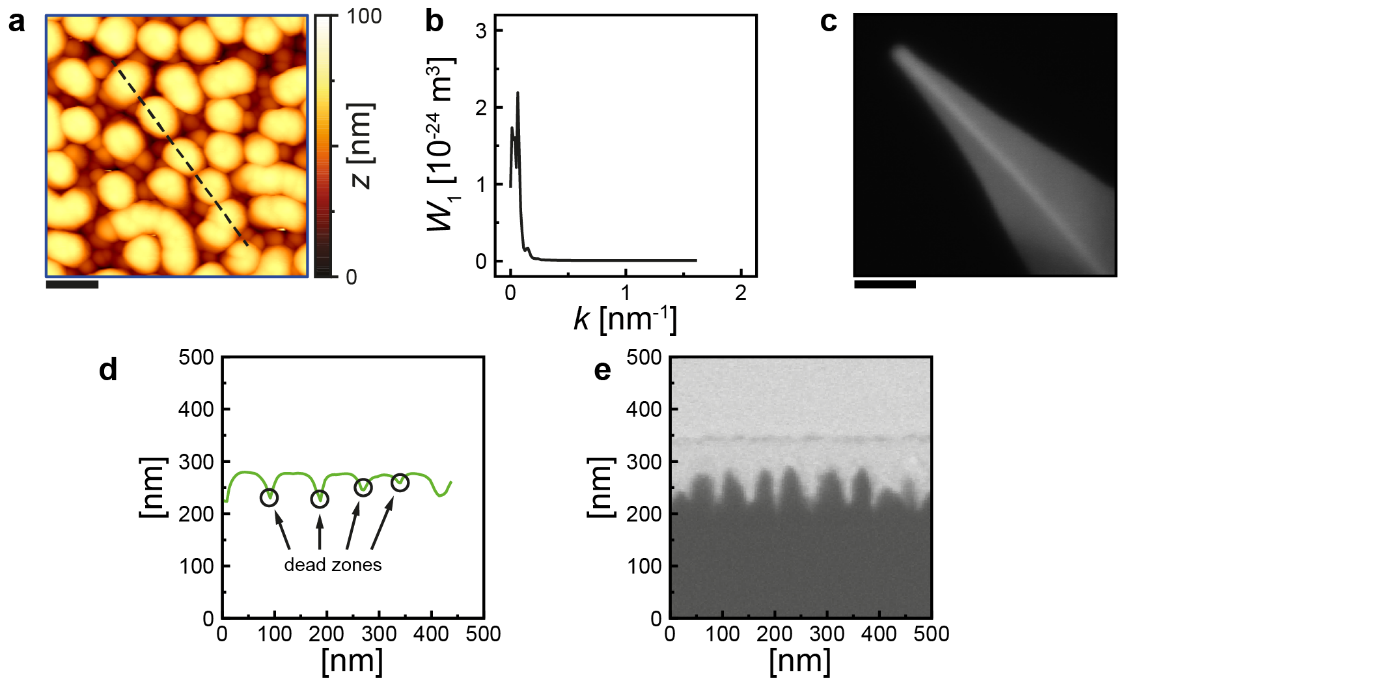


**Figure S7. AFM scan measurement of the nanoengineered surface.** a) Atomic force microscopy (AFM) height scan of the nanoengineered surface. b) Power spectral density of a). c) Micrograph image of the AFM tip after the measurement. d) The profile line of the dashed line in a) indicates the AFM tip's convolution influence with dead zones through sharp trenches^[7]^. (e) FIB-SEM cross-section with sharp trenches deeper than the AFM tip can reach in measurements. Scale bars: (a), (c) 100 nm.

**S6: Radius of mean curvature calculation**

In order to determine the two-dimensional mean curvature radius tensor, *R_ij_* ∈ $\mathbb{R}$*^i^*^×^*^j^*, we follow the elementary differential geometry by Pressley^[8]^. Our implemented approach is given below for completeness. First, we create the two-dimensional tensors, *X_ij_*, *Y_ij_*, *Z_ij_* ∈ $\mathbb{R}$*^i^*^×^*^j^*, where *X_ij_* and *Y_ij_* are the underlying coordinate matrices with Δ*X* = (*X_i,j+1_* - *X_i,j-1_*)/2 = 3.03 nm being the pixel size of the cross-section focused ion beam scanning electron microscopy image, and Δ*Y* = (*Y_i+1,j_* - *Y_i-1,j_*))/2 = 5 nm being the distances between two cross-section images. For given indices, the point *M* on the surface topography *S*(*x*,*y*,*z*) has a height value that is stored in *Z_ij_* (see Figure 2c). The goal is to calculate *R_ij_* using the components of the first and second fundamental form, with the partial first and second derivatives of *Z_ij_*:

1. Calculation of the partial derivatives of *Z_ij_* with respect to *X_ij_* and *Y_ij_* using finite differences:

$\frac{\partial Z_{ij}}{\partial X_{ij}}\approx\frac{Z_{i,j+1}-Z_{i,j-1}}{2\Delta X}$ (S1)

$\frac{\partial Z_{ij}}{\partial Y_{ij}}\approx\frac{Z_{i+1,j}-Z_{i-1,j}}{2\Delta Y}$ (S2)

1. Calculation of the second partial derivatives of *Z_ij_* with respect to *X_ij_* and *Y_ij_* using finite differences:

$\frac{\partial^{2}Z_{ij}}{\partial X_{ij}^{2}}\approx\frac{Z_{i,j+1}-2Z_{i,j}+Z_{i,j-1}}{{\Delta X}^{2}}$ (S3)

$\frac{\partial^{2}Z_{ij}}{\partial Y_{ij}^{2}}\approx\frac{Z_{i+1,j}-2Z_{i,j}+Z_{i-1,j}}{{\Delta Y}^{2}}$ (S4)

$\frac{\partial^{2}Z_{ij}}{\partial X_{ij}\partial Y_{ij}}\approx\frac{Z_{i+1,j+1}-Z_{i-1,j+1}-Z_{i+1,j-1}+Z_{i-1,j-1}}{4\Delta X\Delta Y}$ (S5)

1. Calculation of the components of the first fundamental form:

$E=1+\left( \frac{\partial Z_{ij}}{\partial X_{ij}} \right)^{2}$ (S6)

$F= \frac{\partial Z_{ij}}{\partial X_{ij}}\frac{\partial Z_{ij}}{\partial Y_{ij}}$ (S7)

$G=1+\left( \frac{\partial Z_{ij}}{\partial Y_{ij}} \right)^{2}$ (S8)

1. Calculation of the components of the second fundamental form:

$L={\frac{\partial^{2}Z_{ij}}{\partial X_{ij}^{2}}}/{\sqrt{\left( \frac{\partial Z_{ij}}{\partial X_{ij}} \right)^{2}+\left( \frac{\partial Z_{ij}}{\partial Y_{ij}} \right)^{2}+1}}$ (S9)

$M={\frac{\partial^{2}Z_{ij}}{\partial X_{ij}\partial Y_{ij}}}/{\sqrt{\left( \frac{\partial Z_{ij}}{\partial X_{ij}} \right)^{2}+\left( \frac{\partial Z_{ij}}{\partial Y_{ij}} \right)^{2}+1}}$ (S10)

$N={\frac{\partial^{2}Z_{ij}}{\partial Y_{ij}^{2}}}/{\sqrt{\left( \frac{\partial Z_{ij}}{\partial X_{ij}} \right)^{2}+\left( \frac{\partial Z_{ij}}{\partial Y_{ij}} \right)^{2}+1}}$ (S11)

1. Calculation of the mean curvature radius *R_ij_*:

$R_{ij}=\frac{2(F^{2}-EG)}{LG-2MF+NE}$ (S12)

This is used to calculate the geometric factor *f_ij_* ∈ $\mathbb{R}$*^i^*^×^*^j^* (Section S7, Supporting Information).

**S7: Influence of the radius of mean curvature on the geometric factor *f***

The heterogeneous nucleation rate, *J*, in classical nucleation theory is described through,

$J=J_{0}\exp\left( -\frac{E_{a}}{k_{B}T} \right)\exp\left( -\frac{\Delta G^{*}}{k_{B}T} \right)$ (S13)

where *J*_0_ is a pre-factor that is determined by geometric factors and material-dependent parameters (e.g., density), *E_a_* is the kinetic nucleation barrier, Δ*G** is the heterogeneous thermodynamic nucleation barrier, *k*_B_ is the Boltzmann constant, and *T* is the temperature^[6]^. The kinetic barrier *E_a_* accounts for rates of diffusion and desolvation. The combination of the pre-factor and the kinetic barrier is *J*_kin_ = *J*_0_ $\exp\left( -\frac{E_{a}}{k_{B}T} \right)$, which depends only weakly on the interface mean curvature radius *R*, the supersaturation *σ*, the temperature *T*, and the number density of ions adsorbed at the interface^[9]^. Δ*G** on a surface *S* is defined as

$\Delta G^{*}=f\left( \theta,R/{r^{*}} \right)\Delta G_{H}^{*}=f\left( \theta,R/{r^{*}} \right)\frac{F\omega^{2}\gamma^{3}}{\sigma^{2}k_{B}^{2}T^{2}}$ (S14)

where $\Delta G_{H}^{*}$ is the homogeneous free energy barrier to nucleation, *f* ∈ [0,1] is a geometric factor that depends on the contact angle *θ* of the nucleating phase on the interface, the critical radius of nucleation *r**, and the mean curvature radius *R* of *S*^[10,11]^. $\Delta G_{H}^{*}$ depends on the shape of the nucleating phase, which is quantified by *F* (*F* = 19.71 for a calcite rhombohedron on the (012) plane), the molecular volume *ω* (for calcite: 6.13 · 10^-23^ cm^3^ per molecule), the interfacial energy *γ* (for calcite in solution: 109 mJ m^-2^), and the supersaturation *σ* which is defined as

$\sigma=\ln\left( \frac{a_{Ca^{2+}}\cdot a_{CO_{3}^{2-}}}{K_{sp}} \right)$ (S15)

where *a_i_* is the activity of the ions *i* and *K*_sp_ is the solubility product of the forming phase (calcite: *K*_sp_ = 10^-8.48^ at 25 °C). For a concave surface, see **Figure S8**a, we can define *f* as

$f\left( \theta,R/{r^{*}} \right)=\frac{1}{2}\left\{ 1-\left( \frac{1+\cos\left( \theta\right)\frac{R}{r^{*}}}{g} \right)^{3}- \left( \frac{R}{r^{*}} \right)^{3}\left[ 2-3\left( \frac{\frac{R}{r^{*}}+\cos\left( \theta\right)}{g} \right)+\left( \frac{\frac{R}{r^{*}}+\cos\left( \theta\right)}{g} \right)^{3} \right]-3\cos\left( \theta\right)\left( \frac{R}{r^{*}} \right)^{2}\left( 1-\frac{\frac{R}{r^{*}}+\cos\left( \theta\right)}{g} \right) \right\}$ (S16)

where $g=\sqrt{1+\left( \frac{R}{r^{*}} \right)^{2}+2\cos\left( \theta\right)\frac{R}{r^{*}}}$, and for a convex surface, see Figure S8b, *f* is

$f\left( \theta,R/{r^{*}} \right)=\frac{1}{2}\left\{ 1+\left( \frac{1-\cos\left( \theta\right)\frac{R}{r^{*}}}{g} \right)^{3}+ \left( \frac{R}{r^{*}} \right)^{3}\left[ 2-3\left( \frac{\frac{R}{r^{*}}-\cos\left( \theta\right)}{g} \right)+\left( \frac{\frac{R}{r^{*}}-\cos\left( \theta\right)}{g} \right)^{3} \right]-3\cos\left( \theta\right)\left( \frac{R}{r^{*}} \right)^{2}\left( 1-\frac{\frac{R}{r^{*}}-\cos\left( \theta\right)}{g} \right) \right\}$ (S17)

For smooth surfaces, *R*→∞ and therefore f(*θ*,*R*/*r**) → f(*θ*) = ¼ (1-cos(*θ*))^2^ (2+cos(*θ*)).

**
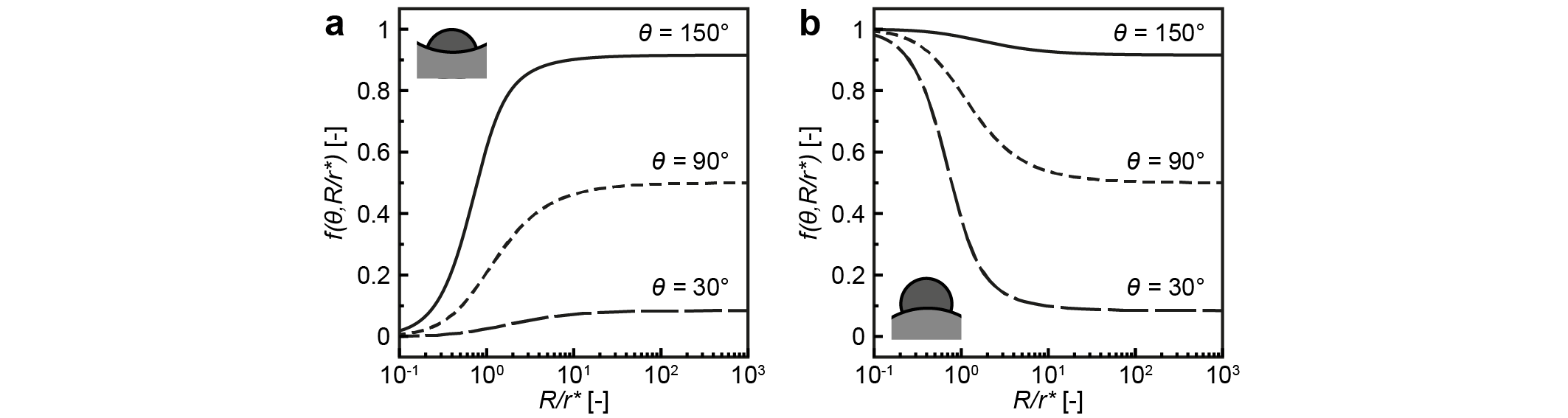
**

**Figure S8. Influence of curvature on the geometric factor f.** a) For concave surfaces. b) For convex surfaces.

**S8: Smooth *f* map analysis**


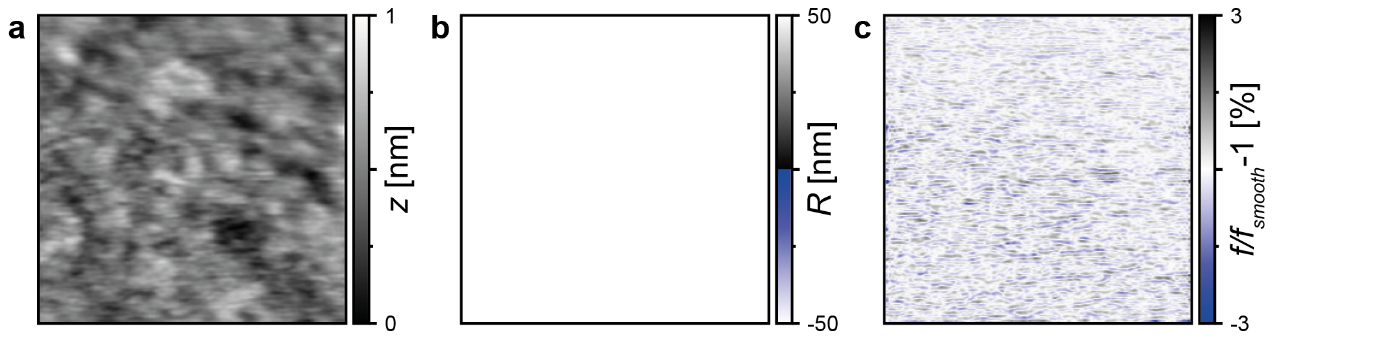


**Figure S9. Surface mapping analysis for the smooth surface.** a) AFM height scan of a smooth surface. b) Computed mean curvature radius map for the height scan with all R values being outside the relevant range of -50 nm to 50 nm. c) Computed geometric factor map for the smooth surfaces. Scan size: 500×500 nm^2^.

**S9: Nucleation across a surface with a distribution of radii of mean curvature**

**S9.1: Surface area calculation**

In order to determine the true surface area, we express the topography of our reconstructed nanoengineered surface (see Figure 2b and c) and our smooth AFM scan (see Figure S1a and e) using the two-dimensional tensors *X_ij_*, *Y_ij_*, *Z_ij_* ∈ $\mathbb{R}$*^i^*^×^*^j^*. Based on this information the surface area tensor *A_ij_* ∈ $\mathbb{R}$*^i^*^×^*^j^* is calculated using surface triangulation for each entry. The sum of *A_ij_* is the true surface area. The Wenzel roughness ratio^[12]^ is defined as the ratio of the true surface area to the projected area. Our nanoengineered surface has a Wenzel roughness ratio, *r*_W_, of 2.45.

**S9.2: Extended classical nucleation theory**

The heterogeneous nucleation rate equation is rewritten as

$\ln\left( J \right)=\ln\left( J_{kin} \right)-B\frac{1}{\sigma^{2}}$ (S18)

Figure 3e shows the linear dependency of ln(*J*_I_) and *σ*^-2^ for a given surface type. For surfaces with chemically homogeneous (*θ* = const) interfaces but different slopes *B*, the only property that differs between the surfaces is their topography and the influence on *f* (the critical radius *r** is not influenced by the topography^[13,14]^).

Sophisticated surface topographies *S*, expressed using the two-dimensional tensors *X_ij_*, *Y_ij_*, *Z_ij_* ∈ $\mathbb{R}$*^i^*^×^*^j^*, have spatially varied *R_ij_* ∈ $\mathbb{R}$*^i^*^×^*^j^* (Section S2, Supporting Information). Based on this information, the surface area tensor *A_ij_* ∈ $\mathbb{R}$*^i^*^×^*^j^* is calculated using surface triangulation for each entry. The sum of *A_ij_* is the true surface area (Section S1, Supporting Information). The heterogeneous nucleation barrier Δ*G** at point *M* on a chemically homogeneous surface *S* follows from Equation S14 to Δ*G**(*M*) = f(*θ*, *R*(*M*)/*r**) $\Delta G_{H}^{*}$. Hence, we can define Δ$G_{ij}^{*}$ ∈ $\mathbb{R}$*^i^*^×^*^j^* as,

$\Delta G_{ij}^{*}=\Delta G_{ij}^{*}\left( \theta,{R_{ij}}/{r^{*}} \right)=f_{ij}\left( \theta,{R_{ij}}/{r^{*}} \right)\Delta G_{H}^{*}$ (S19)

with *f_ij_* ∈ $\mathbb{R}$*^i^*^×^*^j^*. Calculating the nucleation rate in all points on *S*, using Equation S13, results in the nucleation rate tensor *J_ij_* ∈ $\mathbb{R}$*^i^*^×^*^j^*. The overall nucleation rate *J* over a given projected surface area *A* is defined as

$J=\frac{\sum_{i} \sum_{j} J_{ij}A_{ij}}{A}$ (S20)

To evaluate the effect of surface topography on nucleation, we compare a nanoengineered surface with a smooth one. This is done by taking the ratio of the different substrate positions, I and II (see Figure 1b) of equal projected surface areas *A*_I_ = *A*_II_ as

$\frac{J_{I}}{J_{II}}=\frac{J_{kin,I}}{J_{kin,II}}\cdot\frac{\sum_{i} \sum_{j} J_{ij,I}A_{ij,I}}{\sum_{i} \sum_{j} J_{ij,II}A_{ij,II}}$ (S21)

To determine the ratio with this equation, a wetting contact angle *θ* and a critical radius *r** must be chosen. The *J*_kin,I_/*J*_kin,II_ is taken from the linear regressions in Figure 3e since it only weakly depends on *R_ij_* and *σ*. The critical radius *r** is inversely proportional with the supersaturation $\sigma$. Hence, *r** decreases with increasing $\sigma$. **Figure S10**shows for different $\sigma$ that a trajectory with the experimentally observed ratio at that $\sigma$ exists. When crystalline calcite formation is considered, we assume θ = 90°^[15]^ and do not use a shape constant^[16]^, resulting in *r**(*σ* = 4.71) = 2.16 nm and *r**(*σ* = 4.62) = 2.13 nm, which is in between the size range of stable pre-nucleation clusters (≈ 1 nm)^[17]^ and close to critical radius measured particles (≈ 5 nm)^[18]^. The fit does not follow the inverse proportionality of *r** looking at the second decimal. We note that for the values of *σ* presented, the 95% confidence intervals for the nucleation rates in Figure 3e allow for a range of overlapping ratio trajectories and enhance the trend of decreasing *r** for increasing *σ* still possible; see Figure S10c.


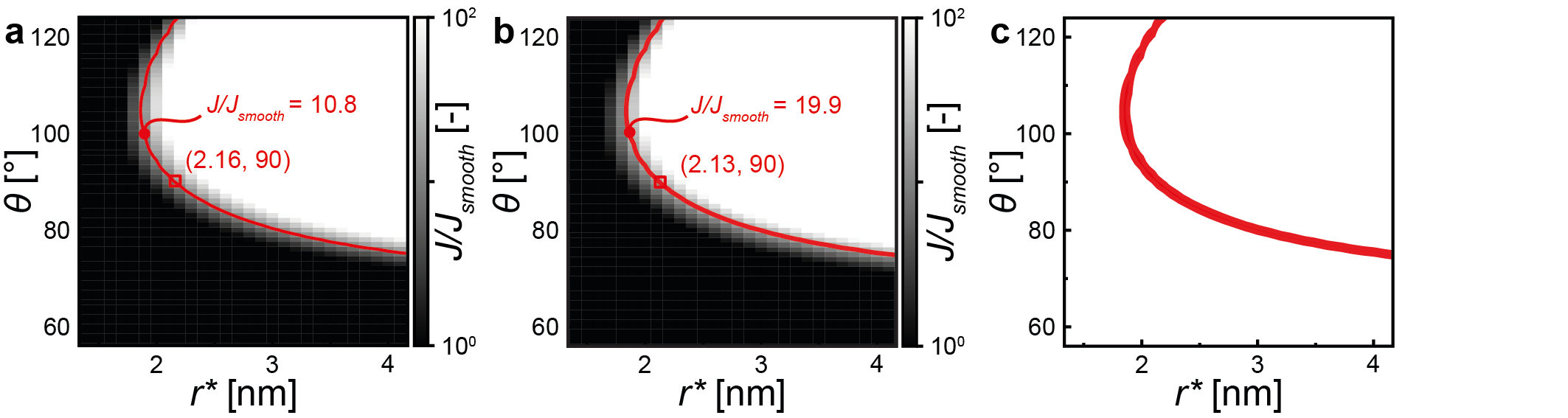


**Figure S10. Nucleation rate dependency on the wetting contact angle θ and the critical radius r*.** The red trajectories indicate the experimentally measured ratio (see Figure 3e) of the supersaturations: a) σ = 4.71 and b) σ = 4.62. Selection of r* and θ for ratio calculation: The new phase of crystalline has no contact angle, and θ = 90° is chosen. r* is calculated by fitting the experimental ratio to the ratio map. It must be in the range between 1 nm (size of pre-nucleation clusters)^[17]^ and 5 nm (stable crystals measured in AFM studies)^[18]^. c) The red region with fitted r*(σ = 4.71) < r* (σ = 4.62) accounts for the linear regression and its confidence band.

**Figure S11** shows *f_ij_*(*θ* = 90°, *r** = 2.13 nm) for different resolutions of nanoengineered surface starting from the resolution of the focused ion beam scanning electron microscopy reconstruction in Figure 2c and the AFM height scan of the smooth surface in Figure S9a. In each coarsening step the resolution changes by eliminating every second row and column of *X_ij_*, *Y_ij_*, *Z_ij_* in the previous resolution and computing *R_ij_*, *A_ij_*, *f_ij_*, $G_{ij}^{*}$, *J_ij_*, and the final *J/J*_R → ∞_ for the current resolution, using the experimental ratio *J*_kin,I_/*J*_kin,II_ for each surface type. It shows for the smooth surface that *J/J*_R → ∞_ is around 1, even if *f_ij_* values vary up to 2.5 % around the *f*_R → ∞_ value. Whereas the ratio for the nanoengineered surface drops, starting from the fitted experimentally observed ratio of 20.6, with each coarsening step approaching *J*_kin,I_/*J*_kin,II_. To obtain the experimentally observed ratio, a new fit is necessary; hence, based on the resolution of *S*, the fit of *θ* and *r** changes. After two coarsening steps, there is no fit for *θ* and *r** to the experimentally observed ratio (see Figure S10), showing that a minimum surface resolution exists for successful fitting of theory and experiment.


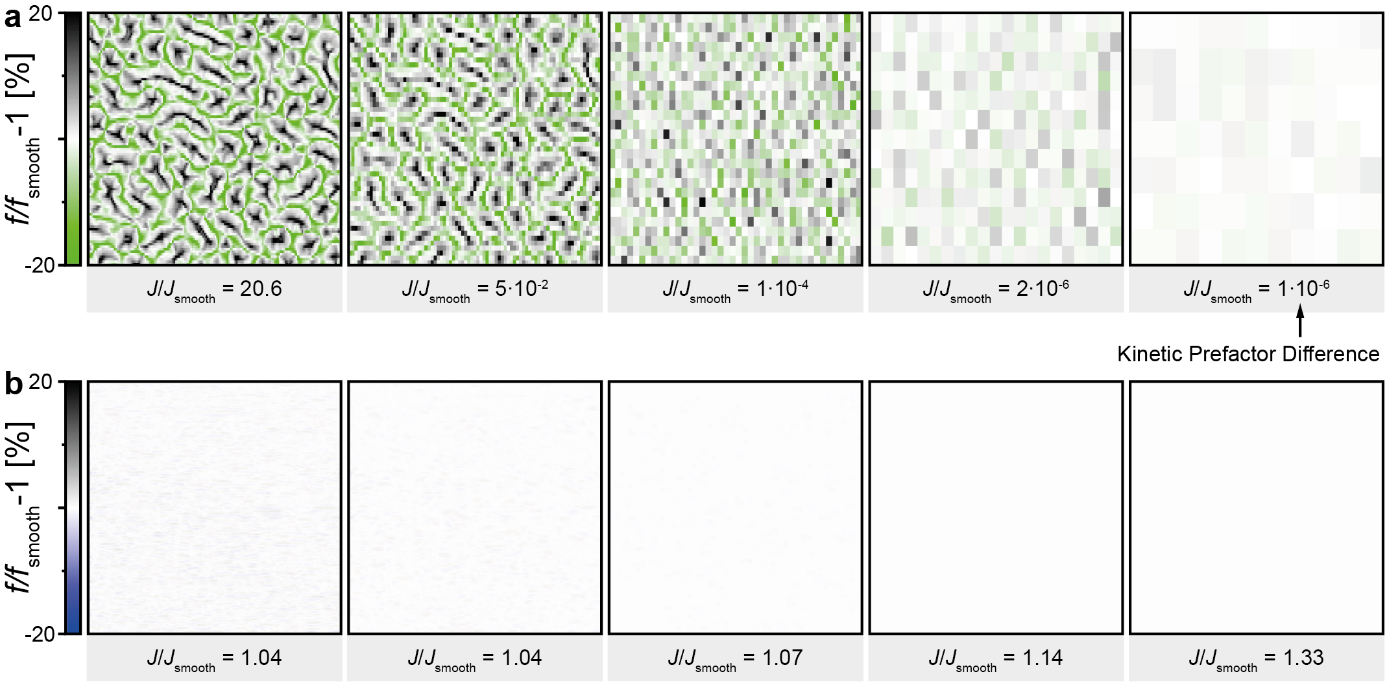


**Figure S11. Coarsening effect on the computed nucleation rate ratio.** The maps of spatial geometric factors f_ij_ and the corresponding computed nucleation rate ratio J/J_smooth_, coarsened in several steps. In one step the maps are coarsened by computing R_ij_ and f_ij_ with every second height value of the reconstructions in the previous step. a) Nanoengineered surface using the micrograph reconstruction of the topography; b) Smooth surface using the AFM height scan. The variation of f_ij_ for the smooth surface is one magnitude lower than the range of -20 % to 20 %. Thus, the maps appear white. Size: 500×500 nm^2^.

**Figure S12** shows that a theoretical scan resolution exists that a coarsening step does not affect the computed nucleation rate ratio.


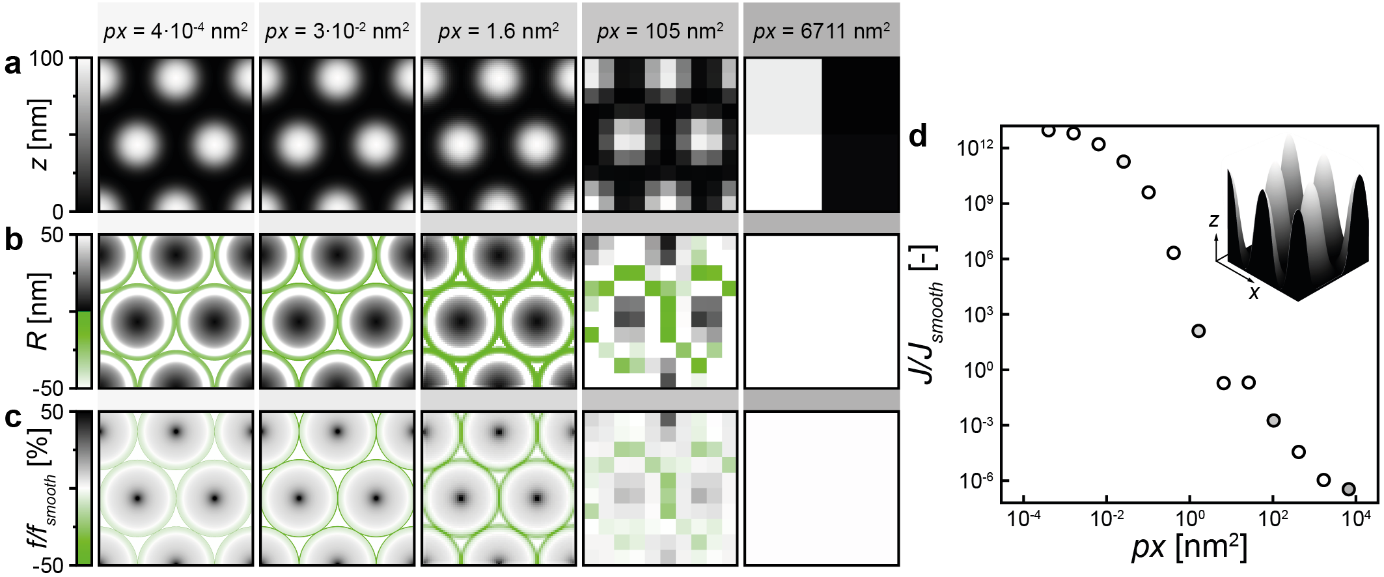


**Figure S12. Theoretical surface (100×100 nm^2^) analysis.** Nanotopography with a diameter of 25 nm, spacing of 50 nm, and height of 80 nm. a) Height map. b) Mean curvature radii map. c) f difference to smooth case map (using r* = 2.13 nm, and θ = 90°). From left to right, the maps are coarsened. The calculated ratios over the pixel size px are plotted in d). At small px the change of ratios through refinement is neglectable. This resolution is way below the capabilities of measurement tools and time. This analysis explains the phenomena qualitatively by picking the parameters r* = 2.13 nm and θ = 90°. One would pick different parameters for different surface reconstruction resolutions to match the explanation with the experimental results. There must be a minimum scan resolution. If the scan point density is too low, the experimental ratio cannot be reached in the intervals r*: 1-5 nm, and θ: 40-130°.

**S10:** **Nucleation behavior at low supersaturation conditions**


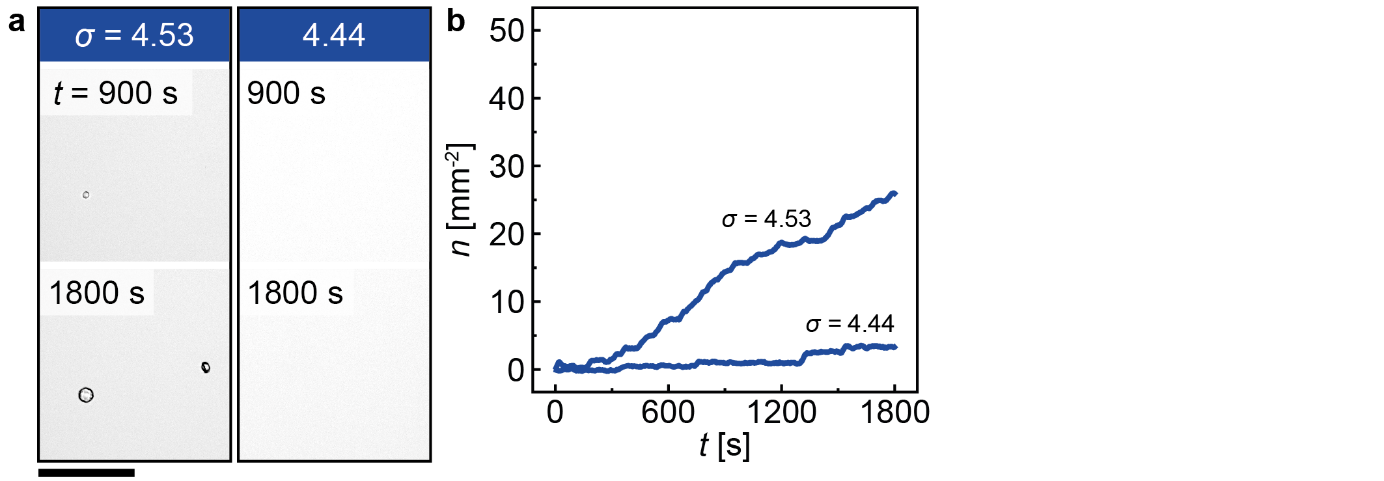


**Figure S13. Nucleation rate evaluation at low supersaturation conditions.** a) Pairs of experimental images with increasing supersaturations for smooth surfaces. The first image shows the experiment at t = 900 s. The second image shows the experiment at the end of the experiment at t = 1800 s. b) Plot of the number of crystallites per area n vs. time t for the smooth surface. Scale bar: 200 μm.

**S11: Depleting through a large number of sites in the mixing zone**

The impact of the changed supersaturation is neglectable for the nucleation rate analysis. At the beginning of every experiment, no crystal growth exists; hence, the system has no depletion of the bulk supersaturation in the observation zone. Every forming growing crystal in the mixing zone, before the field of view is reached, is removing calcium and carbonate ions from the solution^[4]^. For a smooth surface at *σ =* 4.71, around 5000 sites per mm^-2^ exist at the end of the experiment (see Figure 3b) with a mean volume growth rate of 3.6 µm^3^/min (see Figure 5f), and the substrate area is ≈ 25 mm^2^ in the mixing zone before the field of view is reached. This results in a maximal depletion of 1.2 · 10^-5^ mmol/min (calcite density: 2.711 g/cm^3^; calcite molar mass: 100.0869 g/mol). The system is supplied with a calcium and carbonate ion concentration of 1.7 mmol / L at $\dot{V}$ = 500 µL/min, resulting in 8.5 · 10^-4^ mmol/min. The depletion at the end of an experiment accounts for 1.4 % of the supply and lowers the supersaturation by Δ*σ* = 0.019. The depletion at earlier times with fewer sites is lower than this maximal depletion. The impact of the changed supersaturation is negligible for the nucleation rate analysis, given that the linear increasing section of the nucleation curve is present at a significantly lower site density. In Figure 3e, we evaluate only the bottom substrate position, which is the only substrate in contact with the mixing and the observation zone. Hence, it is by design not depleted in the mixing zone for the linear increasing part of its nucleation curve.

**S12: Influence of active defects analysis**

We performed several runs on the same smooth sample to evaluate if there were defects on our surfaces, which are dominant active nucleation sites and might influence the nucleation rate through repeatable nucleation on that defect. Similar analyses have been done previously by Holden et al.^[19]^. **Figure S14** shows the frames with the first 100 detected nucleation sites of five consecutive experimental runs without dismounting the microfluidic stack from the microscope. The system was cleaned with diluted hydrochloric acid to dissolve the crystals, followed by deionized water to remove the acid. Overlapping the segmented images with the location of the nucleation sites in each of the runs, we determine how many of the sites are in common for both experiments (*N*_AB_/[0.5(*N*_A_*+N*_B_)], where *N*_AB_ is the number of sites common to both cycles, *N*_A_ and *N*_B_ represent the total number of nucleation events on cycle A and B). The share of common sites between two runs is, on average, ≈ 5 %, which is very low compared to Holden et al.^[19]^. There is not a single site that nucleated in each of the 5 runs. We conclude that domination of the nucleation rate analysis through defects creating active sites is not present.


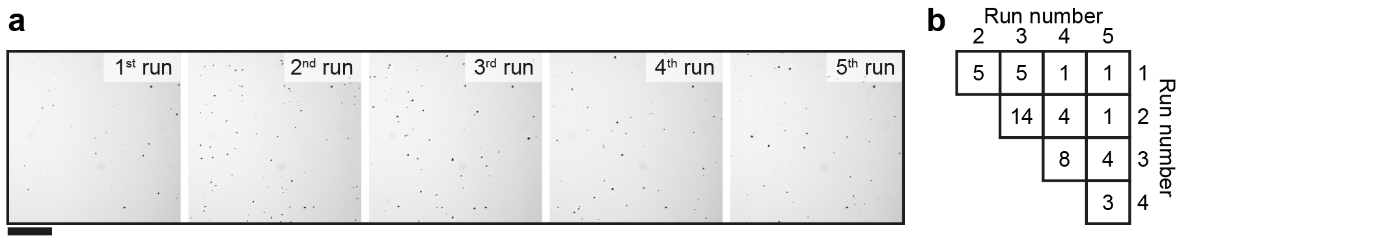


**Figure S14. Influence of surface defects on nucleation.** a) Optical images of a smooth SAM-coated glass surface in 5 different consecutive runs once 100 nucleation sites have been detected by the image analysis. b) Number of sites in common out of the 100 sites between the different runs. Scale bar: 200 µm.

**S13: Projected contact area evolution of TIRF vs. true contact area**


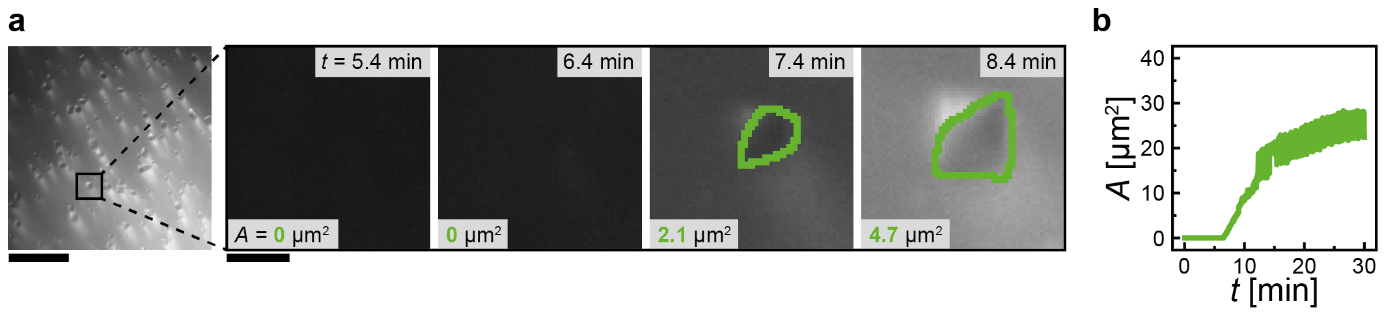


**Figure S15. Projected contact area evolution of crystallite with TIRF.** a) Images sequence at σ = 4.53 of a single calcite crystal nucleating and growing on a nanoengineered surface. b) The evolution of the single crystal's projected contact area vs. time t is plotted in blue. Scale bar 1^st^ column: 20 µm, 2^nd^ – 5^th^ column: 2 μm.


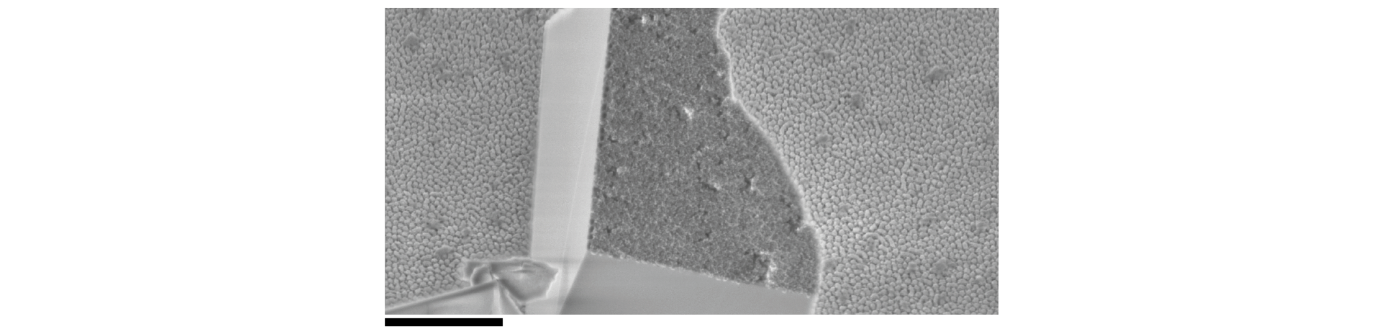


**Figure S16. Micrograph of crystallite contact area on the nanoengineered surface.** The crystallite was pushed off the surface using a micromanipulator with a tungsten tip and flipped after attaching the tip to the crystallite with focused ion beam material deposition. Here, we see that the crystallite does not have a smooth interface like its sidewall. The micrograph does not allow us to assign a specific origin of nucleation. Scale bar: 1 µm.

**S14: Projected area and volume analysis for nanoengineered surfaces at different supersaturations**


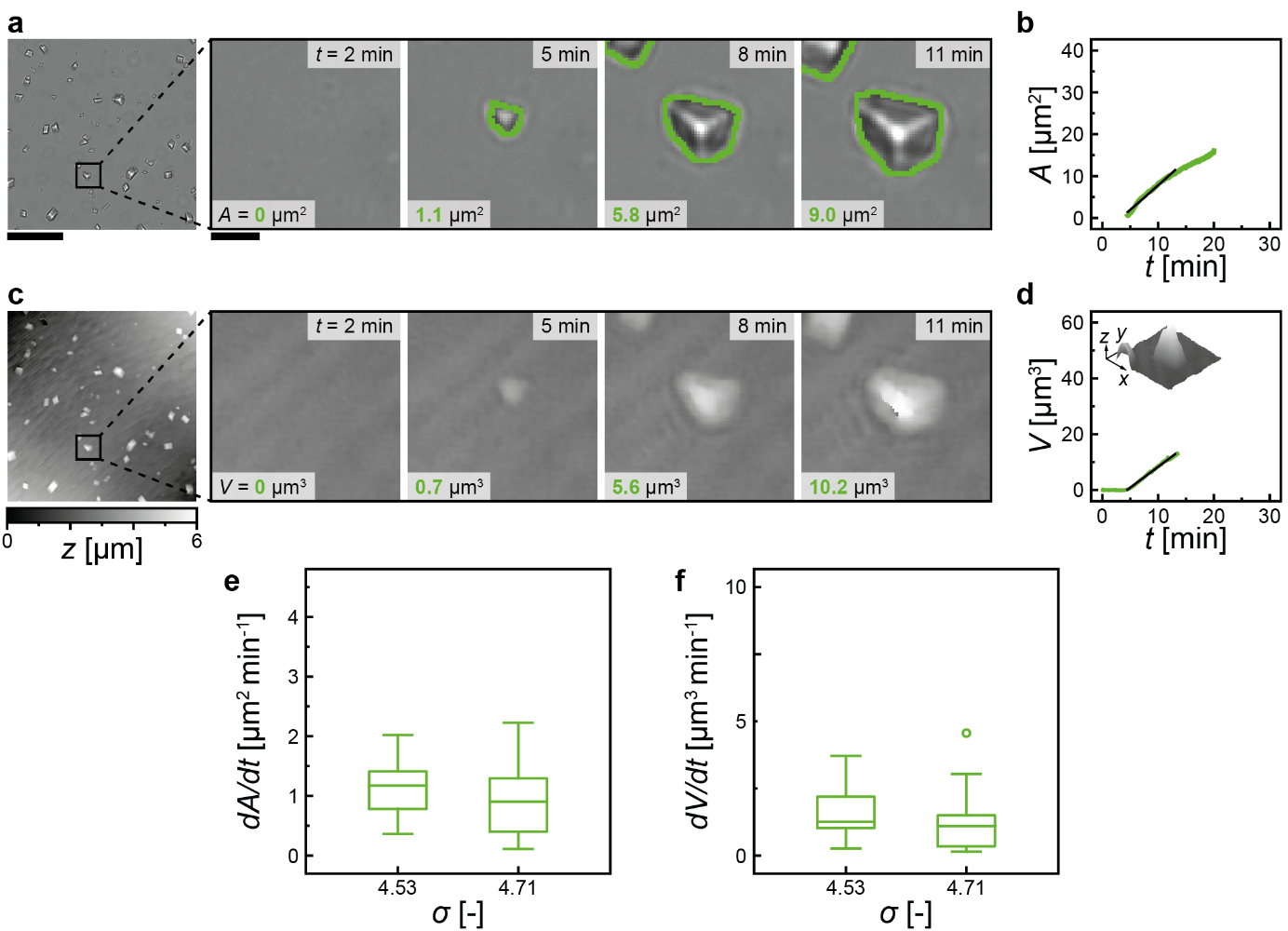


**Figure S17. Single crystal volume and area growth analysis through non-invasive optical measurements.** Simultaneous image sequences at σ = 4.53 of a single calcite crystal nucleating and growing on a nanoengineered surface at substrate position I (see Figure 1b) for a) brightfield imaging and c) digital holographic microscopy (DHM) imaging. The greyscale values of the brightfield images depend on how much light reaches the camera sensor. The greyscale values of the DHM images are height values after phase reconstruction of the acquired hologram. The detected area A and computed volume V in each image is shown. The blue outline of the crystals in a) is calculated using instance segmentation. The evolution of b) the area and d) the volume vs. time t of the single crystal is plotted in blue. The black solid lines are the linear regressions. The inset shows a 3D plot of the crystal after 11 min. The evaluated e) area growth rates and f) volume growth rates are plotted as box plots for nanoengineered (green) surfaces for σ = 4.53 (n=25 single crystals) and σ = 4.71 (n=45 single crystals). The boxes extend from the lower to upper quartile values, with a line at the median, whiskers showing the range of the data, and outliers are those past the end of the whiskers. T-test: e) P-value = 0.05; f) P-value = 0.09. Scale bar: 20 μm; Zoom: 2 μm.

**S15: DHM height validation**


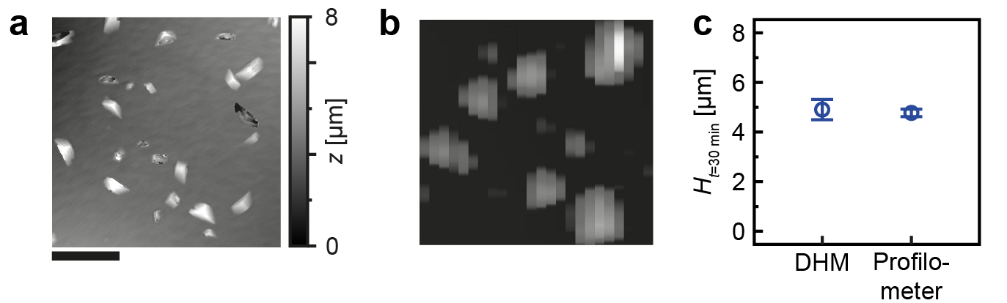


**Figure S18. Validation of DHM height analysis through ex situ profilometer scan.** Height measurement for a smooth surface at σ = 4.71 after t = 30 min with (a) the in situ phase reconstruction of a hologram using the DHM and (b) a 3D map using a profilometer. (c) The mean height and standard error for n=34 crystallites in DHM images and n=106 crystallites in the profilometer 3D maps. T-test: P-value = 0.7. Scale bar: 20 µm.

**Legends for Movies S1 to S3**

**Movie S1.** Nucleation and growth of calcium carbonate sites at supersaturation *σ* = 4.62, comparing smooth and nanoengineered surfaces and the detected number of sites *N* vs. time *t* on the respective surfaces.

**Movie S2.** Image processing of experiments to detect nucleation sites of calcium carbonate.

**Movie S3.** Single crystal analysis of projected area *A* (using instance segmentation) and volume *V* through simultaneous acquisition of optical brightfield microscopy and digital holographic microscopy.

SI References

[1]     T. Casalini, M. Salvalaglio, G. Perale, M. Masi, C. Cavallotti, *J. Phys. Chem. B* **2011**, *115*, 12896.

[2]     Y. A. Le Gouellec, M. Elimelech, *Environ. Eng. Sci.* **2002**, *19*, 387.

[3]     S. Khodaparast, J. Marcos, W. N. Sharratt, G. Tyagi, J. T. Cabral, *Langmuir* **2021**, *37*, 230.

[4]     M. A. Levenstein, Y.-Y. Kim, L. Hunter, C. Anduix-Canto, C. González Niño, S. J. Day, S. Li, W. J. Marchant, P. A. Lee, C. C. Tang, M. Burghammer, F. C. Meldrum, N. Kapur, *Lab Chip* **2020**, *20*, 2954.

[5]     J. Aizenberg, A. J. Black, G. M. Whitesides, *Nature* **1999**, *398*, 495.

[6]     Q. Hu, M. H. Nielsen, C. L. Freeman, L. M. Hamm, J. Tao, J. R. I. Lee, T. Y. J. Han, U. Becker, J. H. Harding, P. M. Dove, J. J. De Yoreo, *Faraday Discuss.* **2012**, *159*, 509.

[7]     B. Voigtländer, *Atomic Force Microscopy*, Springer International Publishing, Cham, **2019**.

[8]     A. N. Pressley, *Elementary Differential Geometry (Springer Undergraduate Mathematics Series)*, Springer, London, **2010**.

[9]     P. M. Winkler, G. Steiner, A. Vrtala, H. Vehkamäki, M. Noppel, K. E. J. Lehtinen, G. P. Reischl, P. E. Wagner, M. Kulmala, *Science* **2008**, *319*, 1374.

[10]    N. H. Fletcher, *J. Chem. Phys.* **1958**, *29*, 572.

[11]    M. Qian, J. Ma, *J. Cryst. Growth* **2012**, *355*, 73.

[12]    R. N. Wenzel, *Ind. Eng. Chem.* **1936**, *28*, 988.

[13]    L. R. Gómez, N. A. García, V. Vitelli, J. Lorenzana, D. A. Vega, *Nat. Commun.* **2015**, *6*, 6856.

[14]    G. Meng, J. Paulose, D. R. Nelson, V. N. Manoharan, *Science* **2014**, *343*, 634.

[15]    A. Koishi, A. Fernandez-Martinez, A. E. S. Van Driessche, L. J. Michot, C. M. Pina, C. Pimentel, B. Lee, G. Montes-Hernandez, *Chem. Mater.* **2019**, *31*, 3340.

[16]    L. M. Hamm, A. J. Giuffre, N. Han, J. Tao, D. Wang, J. J. De Yoreo, P. M. Dove, *Proc Natl Acad Sci USA* **2014**, *111*, 1304.

[17]    D. Gebauer, A. Völkel, H. Cölfen, *Science* **2008**, *322*, 1819.

[18]    Q. Li, A. Fernandez-Martinez, B. Lee, G. A. Waychunas, Y.-S. Jun, *Environ. Sci. Technol.* **2014**, *48*, 5745.

[19]    M. A. Holden, J. M. Campbell, F. C. Meldrum, B. J. Murray, H. K. Christenson, *Proc Natl Acad Sci USA* **2021**, *118*, DOI 10.1073/pnas.2022859118.
